# Behavioral and neural effects of temporoparietal high-definition transcranial direct current stimulation in logopenic variant primary progressive aphasia: a preliminary study

**DOI:** 10.3389/fpsyg.2025.1492447

**Published:** 2025-02-25

**Authors:** Elias D. Granadillo, Mason Fellmeth, Vahab Youssofzadeh, Joseph Heffernan, Priyanka P. Shah-Basak, Sara B. Pillay, Candida Ustine, Peter Kraegel, Shelby Schold, Kimberly D. Mueller, Chrysanthy Ikonomidou, Ozioma Okonkwo, Manoj Raghavan, Jeffrey R. Binder

**Affiliations:** ^1^Institute for Clinical and Translational Research, University of Wisconsin–Madison, Madison, WI, United States; ^2^Neurology, Medical College of Wisconsin, Milwaukee, WI, United States; ^3^Wisconsin Alzheimer's Institute, School of Medicine and Public Health, University of Wisconsin–Madison, Madison, WI, United States; ^4^Department of Communication Sciences and Disorders, University of Wisconsin–Madison, Madison, WI, United States; ^5^Neurology, University of Wisconsin–Madison, Madison, WI, United States; ^6^Division of Geriatrics and Gerontology, Department of Medicine, University of Wisconsin–Madison, Madison, WI, United States

**Keywords:** lvPPA, HD-tDCS, case-series, magnetoencephalography, fMRI, plasticity

## Abstract

**Background:**

High-definition-tDCS (HD-tDCS) is a recent technology that allows for localized cortical stimulation, but has not yet been investigated as an augmentative therapy while targeting the left temporoparietal cortex in logopenic variant PPA (lvPPA). The changes in neuronal oscillatory patterns and resting-state functional connectivity in response to HD-tDCS also remains poorly understood.

**Objective:**

We sought to investigate the effects of HD-tDCS with phonologic-based language training on language, cognition, and resting-state functional connectivity in lvPPA.

**Methods:**

We used a double-blind, within-subject, sham-controlled crossover design with a 4-month between-treatment period in four participants with lvPPA. Participants completed language, cognitive assessments, and imaging with magnetoencephalography (MEG) and resting-state functional MRI (fMRI) prior to treatment with either anodal HD-tDCS or sham targeting the left supramarginal gyrus over 10 sessions. Language and cognitive assessments, MEG, and fMRI were repeated after the final session and at 2 months follow-up. Preliminary data on efficacy was evaluated based on relative changes from baseline in language and cognitive scores. Language measures included metrics derived from spontaneous speech from picture description. Changes in resting-state functional connectivity within the phonological network were analyzed using fMRI. Magnitudes of source-level evoked responses and hemispheric laterality indices from language task-based MEG were used to assess changes in cortical engagement induced by HD-tDCS.

**Results:**

All four participants were retained across the 4-month between-treatment period, with satisfactory blinding of participants and investigators throughout the study. Anodal HD-tDCS was well tolerated with a side effect profile that did not extend past the immediate treatment period. No benefit of HD-tDCS over sham on language and cognitive measures was observed in this small sample. Functional imaging results using MEG and fMRI indicated an excitatory effect of anodal HD-tDCS compared to sham and suggested that greater temporoparietal activation and connectivity was positively associated with language outcomes.

**Conclusion:**

Anodal HD-tDCS to the inferior parietal cortex combined with language training appears feasible and well tolerated in participants with lvPPA. Language outcomes may be explained by regression to the mean, and to a lesser degree, by ceiling effects and differences in baseline disease severity. The intervention has apparent temporoparietal correlates, and its clinical efficacy should be further studied in larger trials.

**Clinical trial registration:**

ClinicalTrials.gov, Number NCT03805659.

## Introduction

Primary progressive aphasia (PPA) is an insidious neurodegenerative disorder that causes a continuous decline in language function that can have a deleterious impact on an individual’s autonomy and quality of life. The current consensus for the characterization of the three classified unique subtypes of PPA are: non-fluent/agrammatic variant PPA (nfvPPA), semantic variant PPA (svPPA), and logopenic variant PPA (lvPPA) (Gorno-Tempini et al., 2011; Mesulam, 1982). These neurodegenerative aphasia syndromes most commonly present with frontotemporal dementia (FTD) and Alzheimer’s disease (AD) as the underlying neuropathologies: FTD in the case of nfvPPA and svPPA, and AD in lvPPA (Mesulam et al., 2014). Although the prevalence of PPA is currently understudied, a 2016 study from the UK estimated a prevalence of approximately 3-4/100,000, while others have associated PPA with approximately 1/3 of frontotemporal lobar degeneration (FTLD) cases (Coyle-Gilchrist et al., 2016; Grossman, 2010). To date, there are no FDA-approved treatments for stopping or reducing the decline seen in individuals with PPA, underscoring the need for investigating effective treatments to increase function and maintain the communicative quality of life.

lvPPA is the most recently classified variant of PPA, and is associated with impaired single-word retrieval, weakened repetition of sentences and phrases, difficulties with spontaneous speech, and impaired phonemic buffer for words. However, motor speech is relatively preserved in these patients with an absence of agrammatism (Gorno-Tempini et al., 2011; Meyer et al., 2015; Montembeault et al., 2018). lvPPA and other less common language disorders account for the initial and most prominent cognitive impairment in approximately 5% of patients with late-onset AD and up to 27% of those with early-onset AD (Mendez et al., 2012). Recently, transcranial direct current stimulation (tDCS) has emerged as a safe and potentially effective treatment option, particularly when given with language training to enhance language outcomes in individuals with PPA (Tippett et al., 2015).

TDCS is a non-invasive form of electrical stimulation delivered via surface electrodes placed on an individual’s scalp. Stimulation is most often applied for 20–30 min during 10-20 daily sessions with a positive anode and a negative cathode electrode placed over different brain regions (Paulus, 2011; Stagg et al., 2018). The benefits of anodal tDCS are thought to be related to its effect on spontaneous neuronal activity and long-term potentiation (Nitsche and Paulus, 2000; Yamada and Sumiyoshi, 2021). Importantly, synaptic changes sufficient to impact behavior only outlast initial stimulation when delivered simultaneously with a behavioral task (Gill et al., 2015; Nozari et al., 2014; Baker et al., 2010; Fertonani et al., 2014; Marangolo et al., 2013; Monti et al., 2008).

High-definition tDCS (HD-tDCS) is a newer technique that allows superior focality of the generated electrical fields (Borckardt et al., 2012). HD-tDCS enhances precision and the ability to focally target specific brain regions compared to conventional tDCS, which in contrast results in diffused stimulation of large sections of the brain (Edwards et al., 2013). The need for precise stimulation in neurocognitive rehabilitation for localized brain injury has been previously speculated (Hogeveen et al., 2016; Shah-Basak et al., 2023) and there are still open questions related to its feasibility, tolerability, and efficacy in patients with PPA. In this current study, we investigated the feasibility and tolerability of HD-tDCS combined with a phonological treatment paradigm, a key deficit in patients with lvPPA (Gorno-Tempini et al., 2008; Henry et al., 2016). We obtained preliminary data on the efficacy of HD-tDCS, focally targeting regions within the posterior perisylvian territory, in particular the inferior parietal nodes that are critically involved in phonological processing to improve phonological outcomes in patients with lvPPA (Newhart et al., 2007; Gorno-Tempini et al., 2004; Pillay et al., 2014a; Montembeault et al., 2018). To compare changes in neural activity with behavioral treatment and HD-tDCS, we used resting-state fMRI. Resting-state magnetoencephalography (MEG) was implemented to study changes in neuronal oscillatory patterns with HD-tDCS. Finally, we also addressed questions related to the duration of the so-called “washout” period or the timing between different stimulation conditions (e.g., anodal vs. sham tDCS; active vs. active-control tDCS) for cross-over designs in PPA.

Most prior studies have used conventional tDCS (Fenner et al., 2019; Hung et al., 2017; McConathey et al., 2017; Roncero et al., 2019; Tsapkini et al., 2014; Zhao et al., 2021). For example, Gervits et al. (2016) used conventional tDCS over the left frontotemporal region in patients with lvPPA and progressive non-fluent aphasia (PNFA) with the explicit aim of targeting the language network without focusing on specific variants. Three prior studies that used HD-tDCS followed a similar framework as those with conventional tDCS of targeting the same area irrespective of variant-specific anatomical correlates. Unal et al. (2020) explored the impact of brain atrophy on tDCS and HD-tDCS current flow across the three PPA variants, but targeted left IFG for all cases. Nissim et al. (2022) that included 12 participants with different variants (8 lvPPA, 2 svPPA, and 2 PNFA) also targeted the frontotemporal region (FT7). Lastly, Shah-Basak et al., 2022, a study in a patient with PNFA targeted anatomically intact left supramarginal gyrus using HD-tDCS based on findings from baseline resting-state MEG.

A few studies using conventional tDCS used a variant-specific target for stimulation. Teichmann et al. (2016) applied conventional tDCS over the anterior temporal lobe and showed that stimulation boosted semantic processing in patients with svPPA. The authors hypothesized that while patients with nfvPPA could respond to modulation of the damaged posteroinferior frontal cortex, the temporoparietal junction would be a promising target for patients with lvPPA. Three prior studies tested this hypothesis in lvPPA by targeting the left temporoparietal cortex using conventional montages. Hung et al. (2017) included a single case of lvPPA within a cohort of 5 (svPPA = 3, AD = 1) and coupled the stimulation with semantic feature training. Roncero et al. (2017) targeted the inferior parietal lobe in 2 patients with lvPPA as part of a larger cohort of 10 (nfvPPA = 6, svPPA = 2) and paired the stimulation with a naming treatment. Anodal stimulation was superior to sham in this study. Roncero et al. (2019) compared frontal and temporoparietal conventional montages in a group of 12 that included four participants with lvPPA (svPPA = 4, nfvPPA = 4); the temporoparietal configuration plus training using a naming treatment appeared to produce longer-lasting effects and greater generalizability to untrained items. Tsapkini et al. (2018) showed tDCS-related improvement in written-naming letter accuracy after stimulation of the left IFG in an oral/written-naming therapy protocol. In this study, patients with nfvPPA, whose main site of atrophy is the stimulated left IFG, seemed to benefit most from the treatment (Coemans et al., 2021). This study also lends support to stimulation approaches targeting the atrophic area in PPA.

In our study, we also implemented a cross-over design with a longer ‘washout’ period. A recent systematic review analyzed the similarities and differences across tDCS studies and examined patient characteristics, stimulation protocols, study design, and their potential influence on treatment effects in PPA (Coemans et al., 2021). This review indicated a multiplicity of washout period lengths across tDCS studies in PPA: 1 week after 5 daily sessions (Wang et al., 2013), 8 weeks after 10 to 15 consecutive sessions (Fenner et al., 2019; Roncero et al., 2019; Tsapkini et al., 2014), and 3 months after 10 to 15 daily sessions (Gervits et al., 2016; McConathey et al., 2017; Themistocleous et al., 2021). Across these studies, the interpretation of tDCS aftereffects was often limited by the possibility of carry-over and/or ceiling effects because of short cross-over periods. For designs involving longer cross-over periods, the interpretation is confounded by increased neurodegeneration during the second cycle of treatment, potentially limiting interpretations of comparing the effects of treatment with the first cycle. In PPA, it is not known whether a longer washout period could help to parse out specific effects of treatments (Coemans et al., 2021), especially in patients with lvPPA. Among the three PPA variants, the slowest rate of decline has been shown to occur in lvPPA (Sebastian et al., 2018). For this reason, a longer washout period might provide a tradeoff between the risk of carry-over effects resulting from shorter ‘wash-out’ periods, and the risk of progressive deterioration stemming from the use of longer ones. To address this question, our study implemented a 4-month cross-over period, the longest compared to any other studies so far using tDCS in patients with lvPPA.

Our study also focused on examining the underlying neural mechanisms of HD-tDCS. Only a few prior studies have attempted to elucidate the neural aftereffects of HD-tDCS treatments in patients with PPA (Coemans et al., 2021). Prior studies have investigated baseline gray matter density and volumes from structural MRI as predictors of treatment response in patients with PPA (Cotelli et al., 2016; de Aguiar et al., 2020; Nissim et al., 2022; Zhao et al., 2021), but focus on functional patterns is limited. To our knowledge, only one study implemented pre-and post-treatment resting-state fMRI (rsfMRI) to evaluate tDCS-induced modulations in functional connectivity (Ficek et al., 2019). Our study examined pre-and post-fMRI to identify the effects of HD-tDCS on language network functional connectivity as well as the influence of the intervention on task-related neuronal oscillatory patterns using MEG. While at least one MEG study demonstrated a distinct spatiotemporal pattern of altered functional connectivity that was unique to each PPA variant (Ranasinghe et al., 2017a), the effect of HD-tDCS on network-specific neuronal dysfunction remains to be elucidated.

The overarching goal of this case-series study is to assess the feasibility and tolerability of our HD-tDCS approach and to provide preliminary behavioral and neural insights into the aftereffects to inform decisions related to the design and execution of future larger-scale clinical trials in PPA.

## Materials and methods

### Participants

Four participants with a diagnosis of lvPPA were enrolled (2 males and 2 females; ages 64-76, mean age = 69, SD = 5.10; Table 1). Participants were diagnosed with lvPPA based on the presence of both classical language impairments and supportive MRI findings (Gorno-Tempini et al., 2011; Mesulam, 2001) as established by a consensus among a behavioral neurologist (EG) with training in early onset and atypical forms of dementia, and both a cognitive neurologist (JB) and a neuropsychologist (SP) with expertise in the assessment and diagnosis of aphasia. All four participants had received a structural MRI within 2.5 years of study participation; those with incompatible implants or other MRI or tDCS related contraindications were excluded from study enrollment. Participants were fluent in English, right-hand dominant, and had no prior history of dyslexia or other learning disorders. All participants had at least 12 years of prior formal education and no prior history of untreated psychiatric disease, severe cognitive, auditory, or visual impairments, seizures, or unstable chronic illnesses. Participants were recruited from the Medical College of Wisconsin Cognitive Neurology Clinic. The study purpose, risks, and procedures were carefully reviewed with each participant. The study was approved by the MCW Institutional Review Board. In accordance with the Declaration of Helsinki, written informed consent was obtained from all participants before they were enrolled in the study.

**Table 1:** Demographic data and neuropsychological testing.

							MoCA			Digit Span f/b/s			Letter/Category Fluency			Picture Naming/Reading Comp.			Word Rhyming		
ID	Age	Sex	Education	Ydx	Ysx	Tx Period	*T*_0_	*T*_1_	*T*_2_	*T*_0_	*T*_1_	*T*_2_	*T*_0_	*T*_1_	*T*_2_	*T*_0_	*T*_1_	*T*_2_	*T*_0_	*T*_1_	*T*_2_
002	69	F	14	1	8	1: Sham	20	12	15	4/4/1	4/6/4	4/3/3	30/13	23/15	13/13	16/13	13/13	15/13	0.90	0.90	0.85
						2: Anodal	15	16	17	4/2/3	4/5/2	2/3/3	21/17	24/21	26/20	13/13	12/12	11/13	0.70	0.85	0.75
004	76	F	12	2	4	1: Anodal	12	7	8	5/6/2	5/4/2	5/6/2	17/13	21/9	13/4	18/12	19/12	16/13	0.80	0.80	0.75
						2: Sham	11	12	10	0/6/4	4/4/4	0/6/2	30/3	25/3	26/4	12/12	20/12	14/12	0.70	0.85	0.95
005	64	M	16	3.5	7	1: Anodal	9	8	14	4/4/3	5/3/4	4/4/2	10/4	13/5	10/6	2/13	3/13	1/12	0.80	0.55	0.60
						2: Sham	12	12	8	6/2/0	3/3/1	3/5/1	8/6	6/7	8/7	3/12	1/13	0/11	0.55	0.65	0.65
006	67	M	12	0.5	1.25	1: Sham	9	7	5	5/2/0	5/0/0	4/0/0	5/2	2/5	0/3	22/12	19/13	16/10	0.60	0.55	0.45
						2: Anodal	3	5	4	4/0/1	4/0/0	3/0/0	0/0	1/2	0/0	13/11	12/13	12/13	0.35	0.45	0.50

		Non-word rhyming			Phonological STM			Trained non-word repetition			Untrained non-word repetition			Trained word and non-word reading			Untrained word and non-word reading		
ID	Tx Period	T_0_	T_1_	T_2_	T_0_	T_1_	T_2_	T_0_	T_1_	T_2_	T_0_	T_1_	T_2_	T_0_	T_1_	T_2_	T_0_	T_1_	T_2_
002	1: Sham	0.92	0.67	0.80	0.73	0.77	0.65	0.69	0.65	0.71	0.67	0.73	0.67	0.86	0.79	0.84	0.74	0.78	0.73
	2: Anodal	0.75	0.89	0.83	0.67	0.58	0.62	0.79	0.79	0.71	0.64	0.74	0.74	0.86	0.91	0.88	0.90	0.93	0.90
004	1: Anodal	0.78	0.67	0.69	0.75	0.85	0.67	0.67	0.75	0.73	0.74	0.72	0.72	0.79	0.79	0.78	0.76	0.80	0.84
	2: Sham	0.81	0.81	0.83	0.77	0.72	0.63	0.73	0.81	0.79	0.63	0.77	0.75	0.75	0.76	0.76	0.75	0.81	0.79
005	1: Anodal	0.64	0.47	0.61	0.82	0.77	0.83	0.56	0.71	0.77	0.56	0.73	0.65	0.68	0.73	0.73	0.73	0.66	0.65
	2: Sham	0.50	0.67	0.47	0.68	0.75	0.77	0.58	0.65	0.75	0.57	0.74	0.60	0.49	0.64	0.63	0.59	0.55	0.71
006	1: Sham	0.56	0.44	0.56	0.53	0.60	0.52	0.44	0.67	0.75	0.54	0.67	0.58	0.38	0.54	0.41	0.44	0.51	0.55
	2: Anodal	0.50	0.47	0.53	0.48	0.48	0.57	0.65	0.63	0.60	0.63	0.73	0.69	0.48	0.51	0.43	0.49	0.44	0.40

Age, participant age in years at the start of study participation; Sex, male (M) or female (F); Education, number of years of formal schooling; Ydx, number of years since initial lvPPA diagnosis at the start of study participation; Ysx, number of years since first language symptoms were noted prior to start of study participation; Tx Period, treatment timeline in which a participant received 10 sessions of either anodal HD-tDCS (anodal) or sham HD-tDCS (sham) paired with language therapy and the assessments conducted before, during, and after; T0, baseline neuropsychological and language assessment prior to treatment with either active HD-tDCS or sham HD-tDCS; T1 = assessment on treatment day 10 of active HD-tDCS or sham HD-tDCS; T2, assessment 2 months after the final treatment session; MoCA, Montreal Cognitive Assessment score out of a maximum score of 30 (Nasreddine et al., 2005); Digit Span f/b/s, digit span forwards, backwards, and series subtest cumulative scores of the Wechsler Adult Intelligence Scale (Benson et al., 2010); Letter/Category Fluency, Delis-Kaplan Executive Function System spontaneous word generation subtest correct response raw scores (Delis et al., 2001); Picture Naming/Reading Comp., Neuropsychological Assessment Battery picture naming raw score (maximum of 31) and reading sample score (maximum of 13) (Stern and White, 2003); Word Rhyming, percentage of correct responses for the choosing of an appropriate rhyming word from two other choices (Pillay et al., 2014); Non-word Rhyming, percentage of correct responses from the choosing of an appropriate rhyming non-word from two other choices (Pillay et al., 2018); Phonological STM, Phonological Short-Term Memory test percentage of correct responses when recalling speech sound information over a 5-s interval (Pillay et al., 2017). Trained Non-word Repetition, non-word repetition task from a list of non-words trained during anodal HD-tDCS or sham sessions, numerical value indicates percentage of correct non-word repetition responses; Untrained Non-word Repetition, percentage of correct responses for a non-word repetition task from a list of untrained non-words; Trained Word and Non-word Reading, percentage of correct responses for a reading task from a list of both words and non-words trained during anodal HD-tDCS or sham; Untrained Word and Non-word Reading, percentage of correct responses for a reading task from a list of untrained words and non-words (Pillay et al., 2017).

### Participant case histories

*Participant 002 (P002)* was a 69-year-old female with 13.5 years of education. She had worked as an administrative assistant until 4 years prior to her lvPPA diagnosis and retired due to age. Approximately 8 years before study participation, she first noted language symptoms, which manifested as word-finding difficulties, an increased frequency of word mispronunciations, and phonemic paraphasias. In addition to language deficits, she had reported increased forgetfulness related to daily tasks and subtle feelings of paranoia, anxiety, and depressed mood. Psychotropic medications taken at the time of the study included aripiprazole and duloxetine. She was independent in her activities of daily living (ADL). Examinations in clinic showed deficits in sentence comprehension and difficulties with repeating long sentences and strings of numbers. Formal neuropsychological testing demonstrated difficulties with word fluency, especially category fluency. She had an overall strong memory performance, but mild declines in executive functions and a significant decline in word finding when compared to formal testing conducted 5 years prior. Volumetric brain MRI using the FDA-cleared Neuroreader software (Aalborg, Denmark) was significant for a left-asymmetric decrease of temporal and parietal lobe volumes, and a low-normal frontal lobe volume compared to normative data. MRI also revealed a mildly decreased arterial spin labeling (ASL) signal within the left anterior and left lateral temporal lobes, as well as left-asymmetric increased diffusivity within the left temporal lobe on mean diffusivity maps.

*Participant 004 (P004)* was a 76-year-old female with 12 years of education, who worked as an office assistant until retiring 4 years prior to the study. Her first language symptoms were noticed 4 years prior to study participation, and she had a diagnosis of lvPPA that was made 2 years prior to the study. Her first symptoms were problems with word-finding and marked pauses in conversation that had been progressively getting worse. She noted frequent word mispronunciations and had difficulties with typing. In addition, she noted some forgetfulness, but without a predilection for either short-or long-term memory. She was independent in all ADLs. Along with the language and cognitive symptoms, she also had experienced a depressed mood, feelings of anxiety, and irritability. She was not taking any psychotropic medications or cognitive enhancers at the time of this study. During examination, she demonstrated non-fluent language with constant stumbles, word-finding pauses, impaired picture naming, and poor repetition. Comprehension, recall, and abstraction appeared to be preserved overall despite language deficits. Formal neuropsychological testing indicated significant impairments in object naming and speech repetition, with generally intact memory and delayed recall. Her processing speed, attention, and single-word reading were average. When compared to normative data, MRI Neuroreader revealed volume loss in the left temporoparietal region with an associated decreased ASL signal. On diffusion tensor imaging (DTI) there was an equivocal regional increase in mean diffusivity within the left temporal lobe white matter, suggestive of a micro-structural abnormality.

*Participant 005 (P005)* was a 64-year-old male with 16 years of education and worked as an engineer until 4 years prior to study participation when he was obligated to retire due to his language deficits. His first symptoms appeared approximately 7 years prior to study participation, and he was diagnosed with lvPPA 3.5 years before. Symptoms initially presented as difficulties with word finding, remembering names, and overall language comprehension. He would later start experiencing memory deficits marked by misplacing objects and repeating questions, in addition to social withdrawal. The medication regimen he was taking at the time of this study included donepezil, memantine, mirtazapine, and sertraline. He was independent in all ADLs. Examination showed speech that was slow and hesitant, punctuated with word finding difficulties. He showed difficulties following complex commands and could not repeat long sentences or number strings. Formal neuropsychological testing revealed an abnormal profile characterized by impairment in most verbally-mediated tasks, including verbal memory, picture naming, word reading, and verbal fluency. On MRI Neuroreader, striking left–right asymmetry involving the temporal and occipital lobes was noted with less volume on the left than the right lobes. Both ASL and prior PET findings suggested hypometabolism within the left temporal and parietal lobes.

*Participant 006 (P006)* was a 67-year-old male with 12 years of education. He had been diagnosed with lvPPA 6 months prior to study participation after approximately 16 months of preceding symptoms. He was previously employed as a truck driver until retiring 4 years prior to the study. Initial symptoms presented as forgetfulness of past conversations and difficulties with word-finding. Additionally, he had been experiencing mood changes, depression, and anxiety. Psychotropic medications taken at the time of this study were buproprion and citalopram. He was independent in all ADLs except for finances, which was managed by his partner. During examination, he showed hesitant speech and word finding difficulties. He had difficulties following complex commands; however, no issues were noted with single word comprehension. On formal neuropsychological evaluation, his cognitive profile was abnormal and notable for below expected performance in the domains of language, memory, processing speed, and executive functioning. His speech was fluent but had a slow rate at times due to word finding hesitations. When speaking, his responses were generally grammatically sound, although often sparse of content. He did not make paraphasic errors when speaking during free conversation. Comprehension was variable, and often with longer and more complicated instructions being harder to understand. He had phonological dyslexia and dysgraphia, as evident by the number of formal paraphasic errors made on real word trials and lexicalization errors on pseudoword trials. MRI Neuroreader showed asymmetrically decreased temporal, parietal and to a lesser extent frontal lobe volumes when compared to normative data. Hippocampal volume on the left appeared smaller compared to the right. Asymmetrically reduced ASL signal was noted in left frontal, temporal, and parietal regions. An old lacunar infarct was also noted in the right cerebellar hemisphere.

### Design

**Figure 1:**
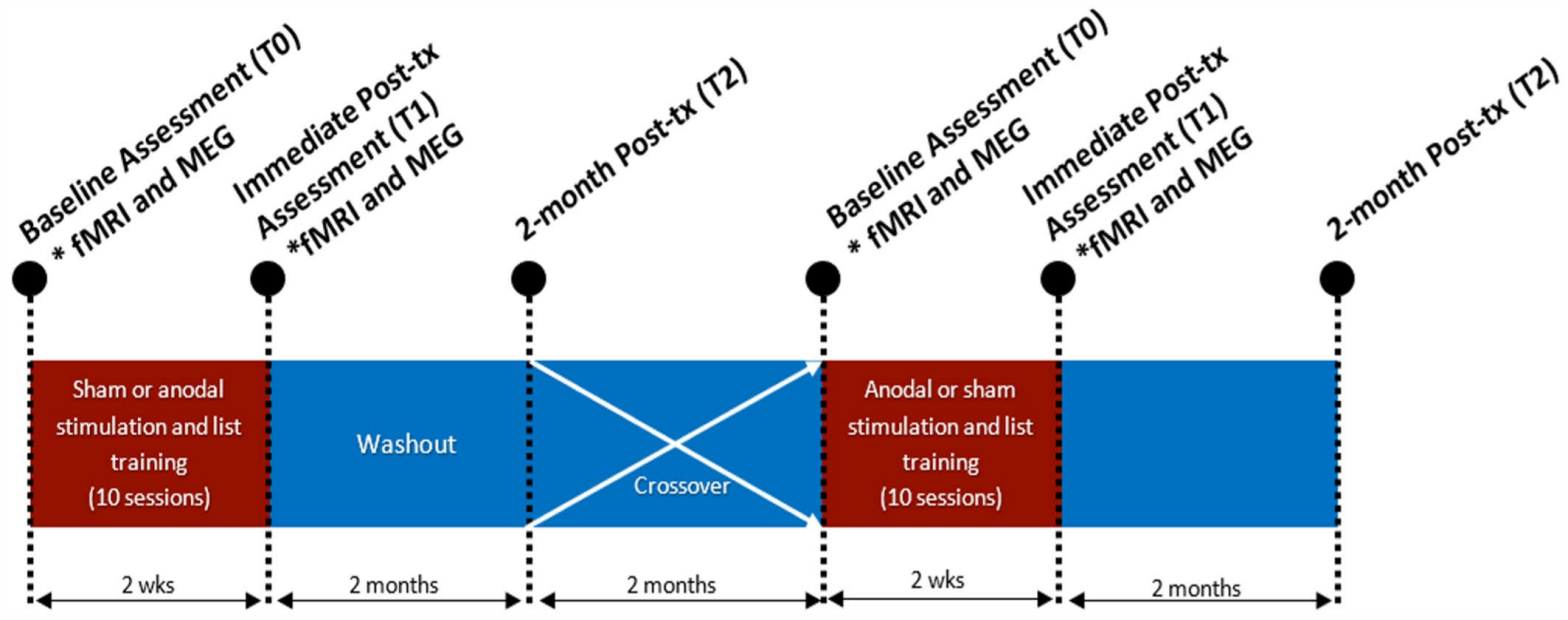
Study design. Participants either received anodal HD-tDCS or sham HD-tDCS over 10 sessions with language and psychometric assessments at baseline (T0), immediately after session 10 (T1) and 2-months after their final treatment session (T2). fMRI and MEG assessments at T0 and T1. Study schedule was repeated after period 1 with a 4-month washout, after which the treatment condition was swapped.

This study utilized a randomized, double-blind, sham-controlled, within-participant crossover design with a 4-month between treatment washout period (Figure 1). Participants were randomly assigned to receive either anodal or sham stimulation in combination with non-word repetition and word/non-word reading language training. Participants completed baseline assessments involving language and cognitive tasks (T0). Following baseline assessments, participants completed 10 daily (M-F) sessions over 2 weeks of anodal or sham in combination with repetition and reading training. Language training lasted 30 min with middle 20-min of training combined with stimulation (training started 5 min prior and ended 5 min after stimulation). Following each treatment session, participants completed stimulation tolerability questionnaires. Participants and experimenters administering stimulation and training were asked to guess the stimulation condition (anodal or sham). At the end of 10 treatment sessions, language and cognitive assessments were completed to assess immediate effects of treatment (T1). Over the course of 4-months following this treatment, no further stimulation or training was provided. Participants completed the language and cognitive assessments at 2 months into the between treatment period to assess maintenance (T2). Participants completed the tolerability questionnaire again to see if there were any longer lasting adverse events. Participants returned at 4-months to complete the second cycle of treatment following the same treatment schedule previously described but with the alternative stimulation condition. At timepoints T0 (baseline) and T1 (immediately after 10 days of treatment) for each treatment cycle, participants underwent fMRI and MEG.

### Language and cognitive testing

The same test battery was administered at baseline (T0) and at two post-stimulation assessment timepoints (T1 and T2; Table 1). To minimize practice effects, alternative forms were used at each timepoint. The test battery included assessments of key language functions that are typically impaired in LvPPA, to track improvements or reduction in decline on these functions with treatment. Standard language tests supplemented by tests from the Language Imaging Lab Aphasia Battery were implemented to assess lexical retrieval, phonological retrieval, and phonological short-term memory (MCW Language Imaging Laboratory; see details below). Picture naming, reading comprehension (Neuropsychological Assessment Battery; Gavett, 2011), letter and category fluency (Denis-Kaplan Executive Function System; Homack et al., 2005), and digit span (Wechsler Intelligence Scale; Ruchinskas, 2019) were completed. Non-word and word rhyme matching and reading, non-word repetition, and phonological short term memory tests from the LIL battery were also administered (see details below). Non-word repetition was the primary outcome measure because it is typically impaired in lvPPA and was partially targeted with training during stimulation. Global cognitive function was tested using the Montreal Cognitive Assessment (Nasreddine et al., 2005a) and digit span tests (Blackburn and Benton, 1957), providing insights into non-language cognitive deficits and performance.

Participants wore a headphone set (Audio-Technica ATH-PDG1) for all auditory tasks and training. Verbal responses were recorded and later transcribed phonetically by a trained team member. Prompts and responses for the rhyme matching and phonological short-term memory tasks were presented and recorded using a touch-sensitive LCD screen. Testing sessions lasted an average of approximately 2.5 h (range 2-4.5 h). Only on one occasion the session lasted 4.5 h and was split across 2 visits given concerns about testing fatigue.

### Test battery

#### Non-word repetition

During the training sessions participants repeated non-words after an auditory presentation (Medler and Binder, 2005). Testing sessions at T0, T1 and T2 consisted of 96 items, 48 non-words that were trained and 48 that remained untrained for testing of generalization. Participants were scored online by the experimenter and were reviewed offline for accuracy by another trained team member.

#### Word and non-word reading

Oral reading of words and non-words was divided into trained and untrained items. Participants were visually presented with words or non-words, varying 3-4 letters in length, in the center of a screen and asked to read aloud the prompted item. Words and non-words were matched on length, bigram and trigram frequency, and orthographic neighborhood density (Medler and Binder, 2005). Within an assessment timepoint, participants were given 160 items to read aloud, 18 of the items were words and 18 were non-words (40 trained words, 40 trained non-words, 40 untrained words, and 40 untrained non-words). After the final assessment and washout period, a new set of 160 items was assigned.

#### Word and non-word rhyme matching

Each trial consisted of a sample word or non-word presented visually on a computer display. Participants are then tasked to select a rhyming item from two choices below the sample (Pillay et al., 2017; Pillay et al., 2014a, 2018). Trials are constructed so that phonologic similarity is uncoupled from orthographic similarity (e.g., does *snow* rhyme with *plow* or *blow*). For word rhyme matching, 20 word triads were tested at T0, T1, and T2 and then switched with a different list of 20 word triads after the washout period. This was similar for non-word triads, however non-word triad lists contained 36 items.

#### Phonological short-term memory

Speech sounds were presented to participants as digital recordings of spoken non-word syllable trains ranging from 1 to 5 syllables in length. After a 5-s maintenance interval, participants were given another syllable train and asked to determine if the pair were the same or different (Pillay et al., 2017). Participants were given 60 trials, with 30 matching and 30 non-matching items, and responded by pressing non-verbal icons representing ‘identical’ or ‘not identical’ on a touch screen. The same items were used between baseline and timepoints T1 and T2 before being switched with a list of 60 new items after the washout period.

#### Picture naming

Using the Neuropsychological Assessment Battery (NAB) picture naming sub-test, participants were shown 31 colored images and asked to name each image within a 10-s response window (Stern and White, 2003). This naming test has shown sufficient reliability in the identification of aphasia across levels of education and a variety of cultures (Sachs et al., 2016).

#### Letter and category fluency

Letter and category fluency subtests of the Delis-Kaplan Executive Function System, participants were asked to name as many items as they could starting with specific letters (BHR or FAS) in the letter fluency test and as many members of specified taxonomic categories (Clothing and Girl’s names or Animals and Boy’s names) as possible in the category fluency test within a 60-s timeframe (Delis et al., 2001). For further analysis, the number of items correctly named was tallied separately for each 15-s interval.

#### Digit span

Wechsler memory scale – Fourth edition digit span assessments (Benson et al., 2010). Scores were based on the maximum number string length for which a correct response could be reliably produced. Number sequences increase in length as long as correct responses are made, and each subtest ends when two consecutive incorrect responses are made.

#### Reading comprehension

Written word and sentence comprehension were assessed using the NAB reading comprehension subtest (Stern and White, 2003). This two-part test requires participants to demonstrate reading comprehension of single words and sentences by choosing a sentence from multiple choices that match a visual prompt.

#### Montreal cognitive assessment

The Montreal Cognitive Assessment (MoCA) was used to monitor general cognitive impairment throughout study participation. The assessment is a one-page 30-point test with 3 different versions (Nasreddine et al., 2005b). One version of the MoCA was used during the first trial period and a different version in the second following washout.

#### Connected speech from picture description

Recordings from the picture description task from the Neuropsychological Assessment Battery (NAB) were transcribed using the Codes for Human Analysis of Transcripts (CHAT) and automatically analyzed by the Computer Language Analysis (CLAN) program (MacWhinney, 1992). Based on previous studies on people with PPA (Ahmed et al., 2012, 2013), we selected the following measures from connected speech: Propositional idea density from connected speech, being a measure of semantic units that contribute meaning (Brown et al., 2008; MacWhinney, 1992), total number of words spoken, ratio of total to unique words, words per minute, and syntactic complexity, or number of verbs per utterance (Mueller et al., 2018).

### Language training

Training protocol was adapted from the Phonological Components Analysis treatment (Leonard et al., 2008; van Hees et al., 2013). Stimuli from the non-word repetition and word/non-word reading tests were used during stimulation sessions. Both word/non-word reading and non-word repetition were trained, each for 15 min. The order of task training was switched daily, for example if reading was trained first on day 1 then on day 2 repetition was trained first, and so on.

Reading training involved hierarchical cuing procedures, a combination of errorful learning and retrieval practice which is deemed to be optimal for individuals with aphasia (Schuchard and Middleton, 2018). We used the following steps: (1) the participant was instructed to read the word or non-word presented on a screen. Feedback was provided as correct or incorrect. When correct, the experimenter immediately moved to the next stimulus. (2) If incorrect, auditory cues for the first and last phonemes were provided by the experimenter, and the participant was asked to read the stimulus again. (3) If incorrect or unable to respond, each phoneme was slowly enunciated by the experimenter, and the patient was asked to read the stimulus one final time before moving to the next item.

For the non-word repetition training, the following steps were utilized: (1) the participant first attempted to repeat the prompt after slow enunciation by the experimenter, then received immediate feedback identifying the response as correct or incorrect. If correct, the experimenter moved to the next item. (2) If incorrect, the experimenter provided the first and last phoneme sounds followed by the pronunciation for the whole task item, and the participant then made a second repetition attempt. (3) If incorrect or unable to respond, each phoneme was slowly enunciated by the experimenter, and the participant was asked to repeat the stimulus one final time before moving to the next item.

To assess the efficacy of blinding procedures and monitor adverse effects related to daily delivery of HD-tDCS, both participants and experimenters (administering stimulation and training) were asked to guess the stimulation condition by choosing from the following three responses: anodal, sham, or unknown. The frequency of correct versus incorrect guesses was calculated based on the ratio of the number of correct responses and total number of incorrect and unknown responses. Post-stimulation adverse event questionnaires were completed by participants after each stimulation session and 4 weeks after the last stimulation session. The participants reported if they experienced any of the following sensations: tingling, scalp pain, burning, pinching, skin irritation, discomfort, warmth, or headache, either at the start of, during, or at the end of stimulation. Participants then rated the sensation on a scale of 1-4 if they experienced it based on the following qualitative modifiers: 1 (mild), 2 (moderate), 3 (significant), 4 (very high).

Change in reading and repetition performance at timepoints T1 and T2 were compared with pre-treatment performance (T0), as done previously in other tDCS language studies in patients with PPA (Sheppard et al., 2022; Tsapkini et al., 2018). As secondary outcome measures, percent changes in accuracy in scores from a psycholinguistic test battery (word and non-word rhyming, phonological short-term memory, picture naming and reading comprehension) and measures from connected speech (picture description) were also compared across T0, T1 and T2 timepoints.

### HD-tDCS

**Figure 2:**
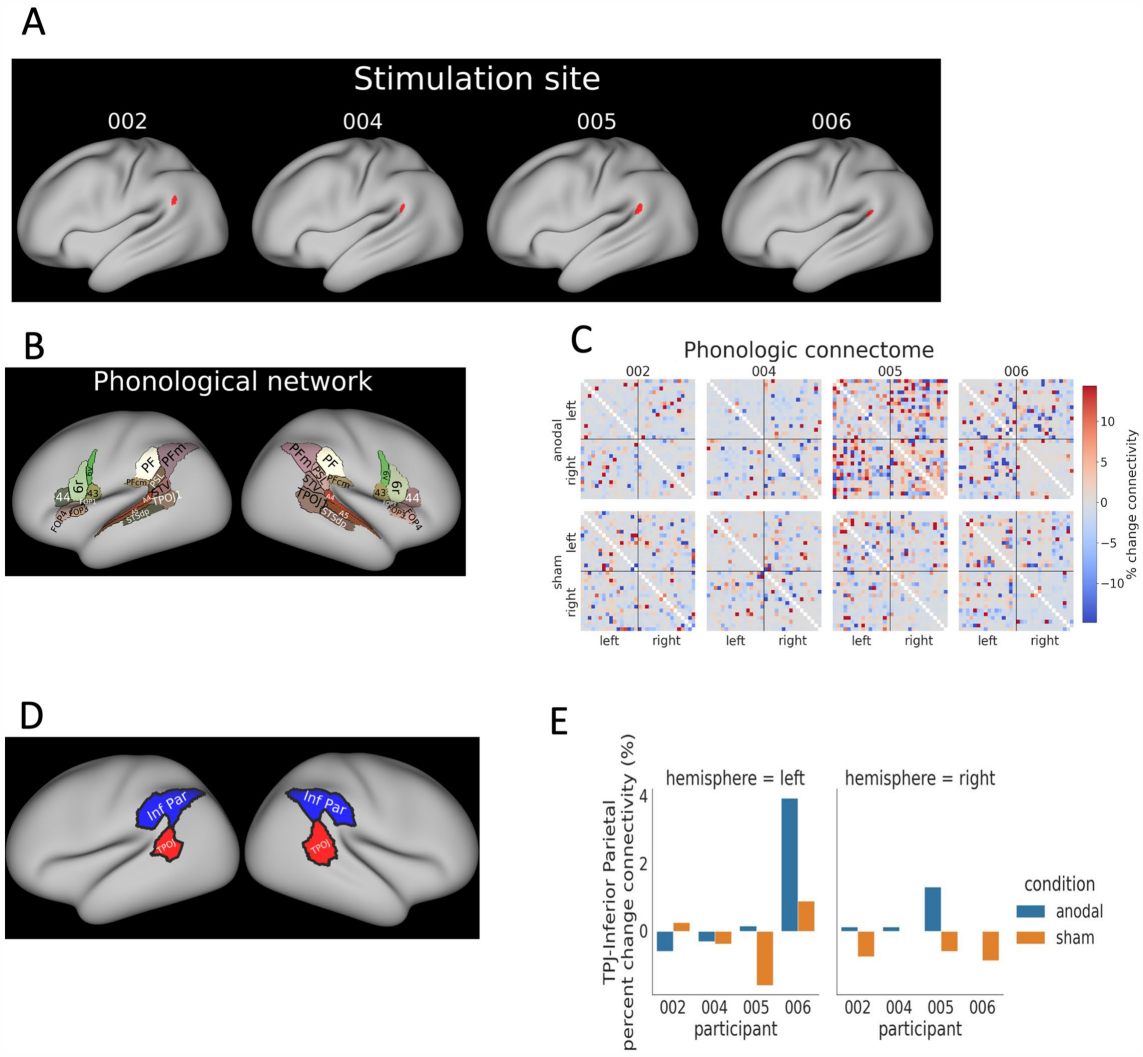
rsfMRI analysis framework. **(A)** Modeled 3D image representation of each respective participant’s left cerebral hemisphere with the red dots indicating the posterior SMG. **(B)** The phonological network was comprised of select Glasser parcels associated with phonological processing. **(C)** The phonologic connectome in % change connectivity for participants during both anodal and sham. **(D)** The inferior parietal and temporoparietal occipital junction (TPOJ) ROIs. **(E)** The intra-hemisphere connectivity between inferior parietal cortex and TPOJ ROIs. Percent change in connectivity is displayed for both the anodal and sham conditions.

HD-tDCS was targeted to the posterior supramarginal gyrus (SMG) (Hickok and Poeppel, 2007a; Pillay et al., 2014a). SMG was localized anatomically in each participant using their T1-weighted MRI scan. In the sagittal view, the posterior point of the planum temporale was first localized to find the most inferior/anterior point of the posterior SMG. The point superior and posterior to this location was marked as the stimulation target. Then a line through the horizontal segment of the sylvian fissure was also used to confirm the target location (Figure 2A). HD-tDCS was delivered using a center-surround 4 × 1 configuration (Datta et al., 2009). A 3D sphere was used to mark the electrode placement of the center electrode, and the 4 return electrodes were placed at a radius of 5 cm from the center electrode.

MRI-based neuronavigation was used to individualize electrode positioning in each participant (Shah-Basak et al., 2020). The T1-weighted images and the sphere indicating the stimulation target were imported into the Brainsight® neuronavigation software (Rogue Research, Quebec, Canada). Participant’s head and brain anatomy were modeled using automatic segmentation algorithms and cortical and surface (3D curvilinear and skin) reconstructions. A disposable latex swim cap was fitted to the participant’s head, and an infrared positioning system together with the neuronavigation software was used to localize the target (or site for the center electrode) on the participant’s scalp. This site was marked in permanent ink directly on the cap. Surrounding return electrodes were also marked on the cap at a radius of 5 cm with 90^o^ angle from one another. The degree of cortical atrophy was not considered when determining electrode placement, and electric field modeling was not completed for the present study.

HD-tDCS was administered using a Soterix “double-blinded” MXN-9 High-Definition stimulator (MXN-9, Soterix Medical Inc). The device was programmed by an unblinded team member prior to each session. The exposed scalp was cleaned with alcohol and electrodes were immersed in conductive gel (HD-GEL™, Soterix Medical), and secured with electrode holders. For active anodal HD-tDCS treatment, a current of 2 mA was administered throughout the 20-min of stimulation. For sham we used a 30-s ramp up to the 2 mA intensity, followed immediately by a 30-s ramp down; this ramp up-down was performed at the beginning and end of the session in order to control for primacy and recency effects (Poreisz et al., 2007; Reckow et al., 2018). The impedance levels were set to be at or below 20 kilo-ohms.

Participants and experimenters who routinely worked with the participants were blinded to the stimulation conditions. An unblinded team member, who had no interaction with the participants and was not involved in HD-tDCS prep, therapy or data scoring, randomized participants to the HD-tDCS condition – anodal stimulation or sham. The order of stimulation condition was counterbalanced across participants.

### fMRI methods

*Data acquisition*: Participants underwent 4 scanning sessions with the same protocol. Data for all sessions were acquired on a 3 T GE SIGNA Premier scanner. A 32-channel Nova head coil was used. T1-weighted (T1w) images were collected with the following parameters: TR = 5.176 ms, TE = 2.264 ms, flip angle = 8°, 0.8 × 0.5 × 0.5 mm voxels. A T2w image with the same voxel dimensions was also acquired to help with surface reconstruction. Simultaneous multi-slice gradient-echo echoplanar BOLD images were collected with the following parameters: SMS factor = 4, TR = 800 ms, TE = 22.3 ms, flip angle = 50°, and 2 × 2 × 2 mm voxels. A pair of spin-echo images with opposite phase encoding directions and the same voxel dimensions as the gradient-echo images were acquired to estimate the B0-non-uniformity. A total of four resting-state runs were collected at each imaging session, each lasting 301.6 s. Participant 004 only had 3 runs in their third session, so we used only 3 runs for all sessions in the analysis for this participant.

#### Preprocessing

fMRI pre-processing was performed using fMRIPrep 23.0.0 (Esteban et al., 2019). All T1w images were corrected for intensity non-uniformity and registered to the first acquired T1w. A reference T1w was created from the T1w images from all four sessions using mri_robust_template (FreeSurfer 7.3.2, Reuter et al., 2010). A reference T2w image was computed after registration of the T2w images again using mri_robust_template. The reference T1w was skullstripped and segmented into cerebrospinal fluid (CSF), white-matter, and gray-matter. Brain surfaces were reconstructed using Freesurfer. Volume-based spatial normalization to MNI152NLin2009cAsym standard space was performed though non-linear registration with antsRegistration (ANTs 2.3.3).

For each session, a displacements field was generated when estimating the B0-non-uniformity map using the two spin-echo echoplanar imaging references with opposing phase encoding directions using topup (Andersson et al., 2003). For each functional run, a reference volume was created using the custom methodology of fMRIPrep. Each run was motion-corrected to their reference volume, corrected for susceptibility distortions using the displacements field, warped to MNI152NLin2009cAsym standard space, and resampled to a 2 mm isotropic grid in a single interpolation step. The preprocessed BOLD images were projected onto fsaverage surface space and resampled to fsLR surface space at 91 k samples (Glasser et al., 2013).

Automatic removal of motion artifacts using independent component analysis (ICA-AROMA; Pruim et al., 2015) was performed on the preprocessed MNI space BOLD time-series after removal of non-steady state volumes and spatial smoothing with an isotropic Gaussian kernel of 6 mm full-width half-maximum. Corresponding “non-aggressively” denoised runs were produced using fsl_regfit. High pass-filtering at 0.1 Hz and further nuisance regression were simultaneously performed on the denoised runs using 3dTproject (Cox, 1996). The nuisance regression included white matter and CSF mean time series as well as quadratic polynomial trends. All resting runs were concatenated within a session to generate a single session run.

#### Phonological connectome

For each participant’s resting fMRI data for each session, a phonological connectome was created using 32 ROIs in the Human Connectome Project (HCP) atlas (Glasser et al., 2016) implicated in phonological processing (Figure 2B) as determined by prior fMRI connectivity analysis during a phonological task (Hickok and Poeppel, 2007; Pillay et al., 2014; Pollack and Ashby, 2018; Vigneau et al., 2006a). BOLD data from each ROI were spatially averaged over time to generate a time series for each ROI. For connectivity estimates, a correlation matrix was computed by comparing each time series to all other time series using Pearson correlation analysis, which was subjected to Fisher‘s r-to-z transformation (Figure 2C). For both anodal and sham tDCS sessions, the percent change in connectivity between the ith and jth ROI was computed as follows: pc_ij_ = ((cond_ij_ – cond_baseline_ij_) / cond_baseline_ij_), where pc is the percent change in connectivity, cond is the tDCS condition (anodal or sham), and cond_baseline is the connectivity at baseline during the first fMRI session, prior to any tDCS.

#### ROI connectivity

In a separate analysis, we also explored percent changes in intrahemispheric connectivity with respect to temporoparietal junction (defined by parcels TPOJ1 and STV from the HCP atlas) and the inferior parietal cortex (defined by PF and PFm parcels; Figure 2D). These ROIs were defined by combining phonological ROIs near the tDCS target.

### MEG analysis

#### Word-recognition task

**Figure 3:**
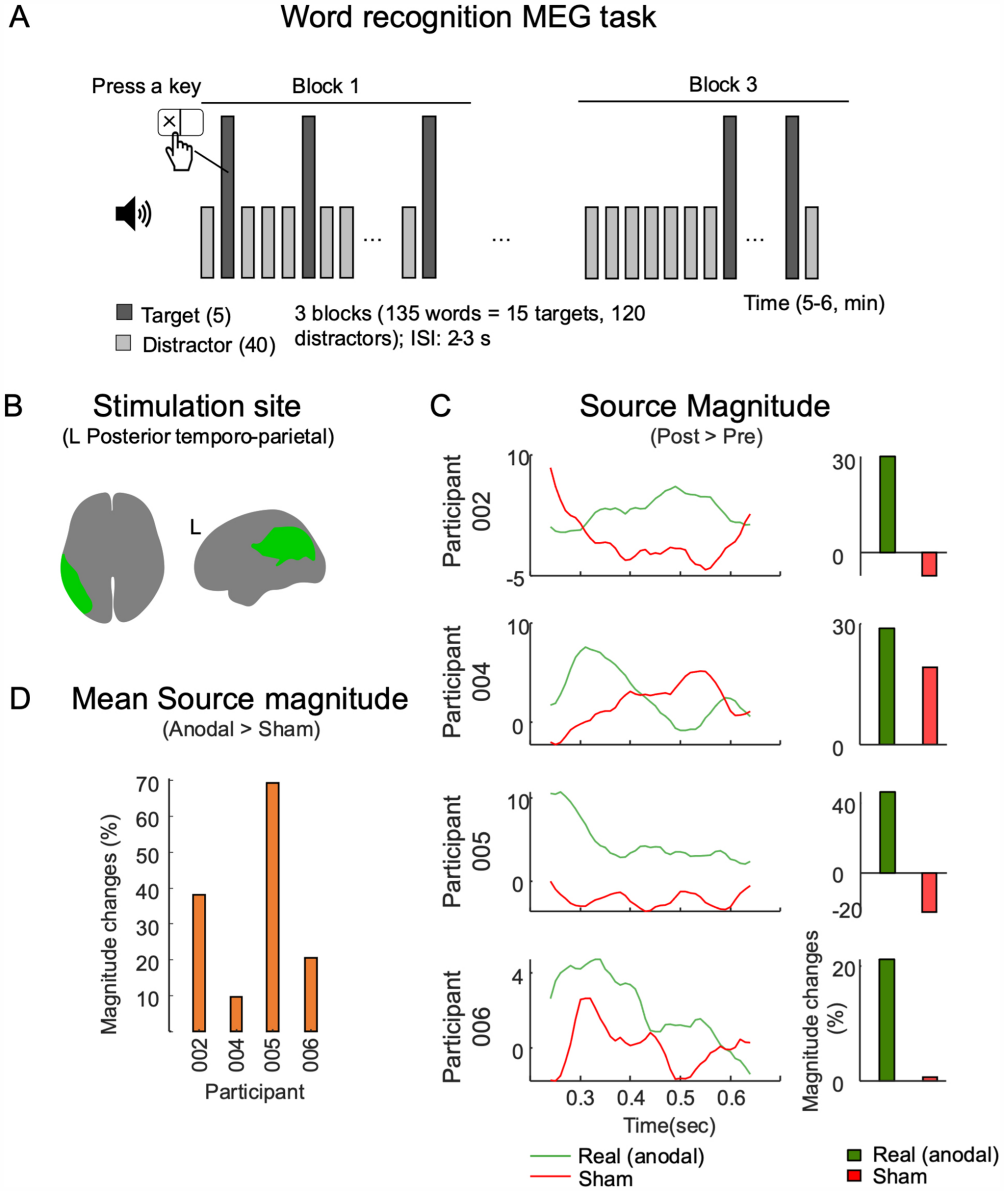
MEG source magnitude near stimulation site. **(A)** The MEG sessions utilized a word-recognition task, which consisted of three blocks of words (5 targets and 40 distractors). These words were presented aurally with a randomized interstimulus interval (ISI) of 2–3 s. **(B)** The temporal–parietal-occipital functional network as mapped in the HCP atlas was used as a reference for examining the evoked cortical responses. **(C)** Source level response analysis was conducted within a 250,650 ms interval after stimulus onsets, and the average difference of response magnitudes (post-session - pre-session) are displayed. **(D)** A comparison of changes in response magnitudes between the anodal and sham conditions.

Degradation of spoken word recognition occurs over time in lvPPA, and the atrophy of temporal and parietal lobes, characteristic of lvPPA, may account for this impaired single word comprehension (Sikora et al., 2021). The language task in the MEG was designed to probe the neural oscillations essential for phonological decoding and lexical retrieval—key to comprehension-during an auditory word recognition task. We hypothesized that this task will allow monitoring of the neural changes induced by tDCS as they relate to single word comprehension. Participants engaged in an auditory word-recognition task (depicted in Figure 3A), distinguishing five target words from 40 distractors, as outlined in prior studies (Papanicolaou et al., 2004; Raghavan et al., 2017; Youssofzadeh and Babajani-Feremi, 2019). The words, read by a native English speaker and presented with a 2–3 s interstimulus interval (ISI), included four monosyllabic (jump, please, drink, good) and one disyllabic word (little), with a frequency ranging from 32 to 194 occurrences per million in the G6-7 corpus (Zeno et al., 1995). The task, divided into three blocks per run, involved 120 distractors and 15 targets, totaling 135 words. Distractor stimuli consisted of familiar common nouns that were unrelated semantically or phonologically to the target words. To maintain engagement, patients were instructed to press a key with their dominant hand when they recognized a target word. Each patient completed two runs of the task across four visits, encompassing both pre-and post-treatment sessions with sham and anodal stimulation.

#### Data acquisition and preprocessing

MEG data were collected using a 306-channel whole-head neuromagnetometer system (Vectorview™, Elekta-Neuromag Ltd., Helsinki, Finland), situated in a magnetically shielded room at Froedtert Hospital, Milwaukee, WI, USA. This system consists of 204 planar gradiometers and 10 magnetometers. Data were recorded at a 2 kHz sampling rate, applying a high-pass filter set at 0.03 Hz. Head localization measurements were conducted before and after each MEG session to monitor head movements. Using MaxFilter software (ver. 2.2, MEGIN Oy, Helsinki, Finland), temporal signal space separation was performed to reduce external magnetic interference, correct signal distortions from head movements, and standardize head positions (Taulu and Simola, 2006). For computational efficiency and to reduce high-frequency artifacts, the MEG data were downsampled to 1 kHz and low-pass filtered at 40 Hz. Signal-space projection methods were utilized to mitigate artifacts related to cardiac and eye movements (Tesche et al., 1995). Furthermore, trials likely affected by head or muscle movements were excluded, as indicated by kurtosis values exceeding 10 and z-scores over 4 (Youssofzadeh et al., 2020).

#### Source analysis of evoked responses

This analysis specifically targeted responses to distractor word stimuli occurring between 250 ms (to control for sensory task responses) and 1 s post-stimulus onset. Trials associated with target word stimuli were excluded to control for motor activations. The MEG data were co-registered with T1-weighted MR images, and source estimation was conducted using a linearly constrained minimum variance beamforming approach (Van Veen et al., 1997). Examination of evoked source power magnitudes focused on areas adjacent to the stimulation sites in the left hemisphere, as shown in Figure 3B. The HCP multi-modal parcellation atlas (MMP 1.0) was employed for regional identification (Glasser et al., 2016). For ROI-based analyses and comparisons, source activations were projected onto the ICBM152 default MRI template surface, spatially smoothed with a 3 mm full width at half-maximum and normalized against a-300 ms pre-stimulus MEG task baseline. Averages from two runs were utilized for interpreting and comparing task activations. Task source magnitudes from both pre-and post-stimulation sessions were contrasted and reported as changes attributable to the intervention treatment in MEG responses.

#### Hemispheric dominance

**Figure 4:**
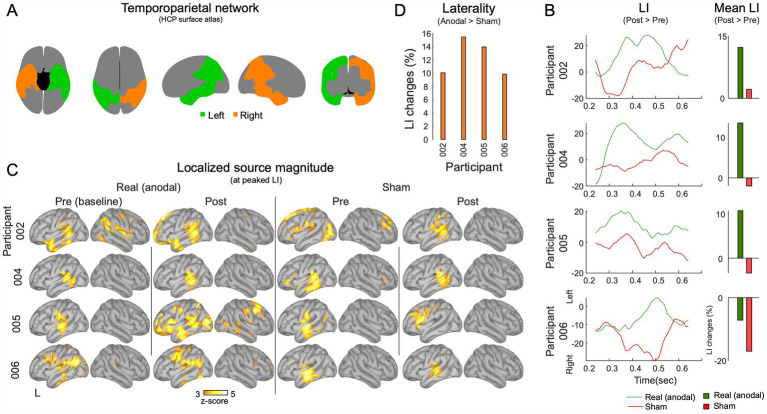
MEG Laterality analysis in the temporoparietal network. **(A)** The temporoparietal functional network. **(B)** The laterality analysis was carried out within the 250–650 ms intervals during the execution of the task. Mean laterality index (LI) values for post-session exceeding pre-session are showcased. **(C)** The source activations at the point in time when the temporoparietal network’s shows peak left laterality. **(D)** A comparison of LI changes between the anodal and sham conditions.

Hemispheric asymmetries in task response source magnitudes within the temporoparietal network were computed within a network including the lateral temporal cortex, temporo-parieto-occipital junction, superior parietal cortex, and inferior parietal cortex, as identified using the HCP atlas (refer to Figure 4A). The primary aim was to examine changes in hemispheric dominance of task-related cortical activations over time, inspired by prior research underscoring the network’s role in language comprehension and processing (Binder et al., 2009; Springer et al., 1999; Vigneau et al., 2006). The laterality index (LI) was calculated as: (L - R)/(L + R), where ‘L’ and ‘R’ represent the source magnitudes in the left and right hemisphere regions of interest, respectively. LIs were derived by counting active vertices that exceeded half-maximum thresholds in both left and right temporoparietal parcels. The analysis compared LI values across pre-and post-stimulation sessions, as well as between sham and anodal conditions. This approach determined potential shifts in hemispheric dominance during auditory language tasks as function of anodal HD-tDCS and language training.

### Statistics

Given the small sample size, we ran within-participant item-level analyses, as previously done in case series studies involving patients with PPA (Hung et al., 2017; Sheppard et al., 2022; Tsapkini et al., 2014). We used statistical methods similar to those used by Sheppard et al. (2022), studying 3 participants with PPA. For the interpretation of changes in language and other cognitive assessments, participant performances were quantified as the percent change between baseline score and either T1 or T2 using: $\frac{Pre-\mathrm{Post}}{Pre}\times 100$ .

To account for potential carryover effects and the effects of neurodegeneration between phases, linguistic outcome measures were compared separately for each phase. The Cochran’s *Q* test was used to determine if the responses at the three time-points (within a treatment period, either anodal or sham stimulation) were significantly different from one another. When significant, Cochran’s *Q* tests were followed up with non-parametric pairwise comparisons using McNemar test with false discovery rate correction (Hung et al., 2017; Sheppard et al., 2022; Tsapkini et al., 2014). To assess differences in percent changes for each test within an individual participant (Sheppard et al., 2022), we ran Wilcoxon-signed rank tests between tDCS conditions (anodal vs. sham) to test if anodal stimulation indeed had an additional beneficial effect compared to behavioral treatment alone. The connected speech measures were analyzed at the individual level in the same way as the trained and untrained items, but given the continuous nature of the data, a paired *t*-test was used.

A group-level paired *t*-test was performed for each unique fMRI node (*N* = 496) between the anodal and sham treatment groups. Multiple comparisons were corrected using the Benjamini-Hochberg false discovery rate correction. A separate group-level paired t-test was performed between the anodal and sham treatment groups (within a treatment cycle) (independent variable) to explore the percent change in fMRI-based connectivity in the inferior parietal and TPJ ROIs across left and right hemispheres (dependent variables). Multiple comparisons (*N* = 2) were corrected using the Benjamini-Hochberg false discovery rate correction All statistical analyses were performed using SPSS version 27.0 (IBM, Armonk NY).

## Results

### Overall blinding and tolerability

Participants responded with “unknown” on 69% of responses on the post-stimulation questionnaire. For the responses that were not “unknown,” participants could distinguish the correct tDCS condition with 44% accuracy. Researchers responded with “unknown” on 30% of responses and with 54% accuracy for correct tDCS conditions. Neither participants’ nor researchers’ accuracy exceeded chance [*X*^2^ (1, *N* = 59) = 0.42, *p* = 0.52]. During period 1 participants guessed with 77% accuracy [*X*^2^ (1, *N* = 13) =3.77, *p* = 0.052] but with only a 12 % accuracy during period 2 [*X*^2^ (1, *N* = 10) =3.6, *p* = 0.057], arguing against learning effects. On tolerability assessments, participants responded that they experienced no side effects for 46% of stimulation sessions (anodal = 45%; sham =48%). For sessions in which participants did report side effects, scalp pain was most common (reported during 30% of anodal and 27.5% of sham stimulation sessions), followed by warmth (reported during 35% of anodal and 15% of sham stimulation sessions), tingling (reported during 15% of anodal and 7.5% of sham stimulation sessions), and burning sensation (reported during 7.5% of anodal and 10% of sham stimulation sessions). On average, magnitude rating of sensations was as follows: mild 44.3% (anodal 44.1%, sham 44.4%) moderate 44.3% (anodal 55.9%, sham 29.6%), and considerable 11.5 % (anodal 0%, sham 25.9%). However, there were no lasting complications or side effects from HD-tDCS at 4-weeks follow-up for anodal or sham conditions. None of the participants withdrew from the study due to tolerability issues.

### Participant 002

#### Trained/untrained treatment items

**Figure 5:**
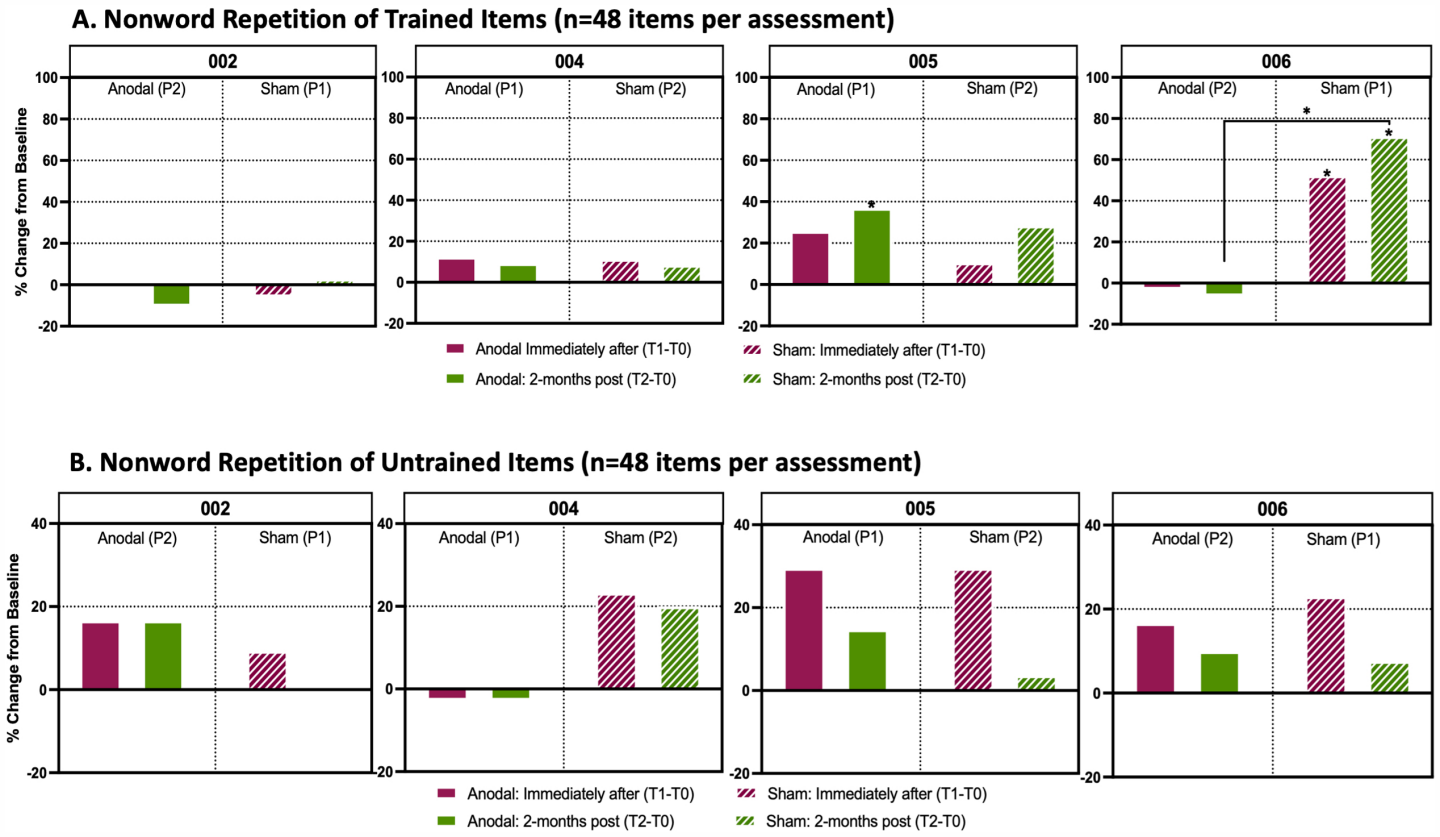
Analysis of trained and untrained repetition and reading tasks. Percentage change from baseline in individual accuracy scores between timepoints in language assessments. **(A)** Non-word repetition task prepared from a list (*n* = 48 items per timepoint assessment) that participants were trained with during active anodal HD-tDCS (Anodal) or sham HD-tDCS (Sham) stimulation sessions. **(B)** Non-word repetition task prepared from a list (n = 48 items per timepoint assessment) that participants were untrained. Solid boxes indicate the change in performance from baseline when participants received anodal stimulation, whereas stripped boxes indicate performance change from baseline when participants received sham treatment. The maroon boxes identify the percent change from baseline immediately after the final stimulation session (T1-T0) and the green boxes show the percent change from baseline 2-months after the final stimulation session (T2-T0). P1 indicates that a participant received Anodal or Sham treatment during the first trial period; P2 indicates that a participant received the respective treatment during the second trial period after a 4-month washout following P1. An asterisk (*) above a box indicates that there was a significant change (*p* < 0.05) in performance between a participant’s baseline score and their assessments either immediately after treatment or 2-months post treatment. These assessments were analyzed using a Cochran Q and post-hoc McNemar test. An asterisk between two treatment periods indicates a significant change from baseline between anodal and sham as determined by a Wilcoxon signed rank test.

**Figure 6:**
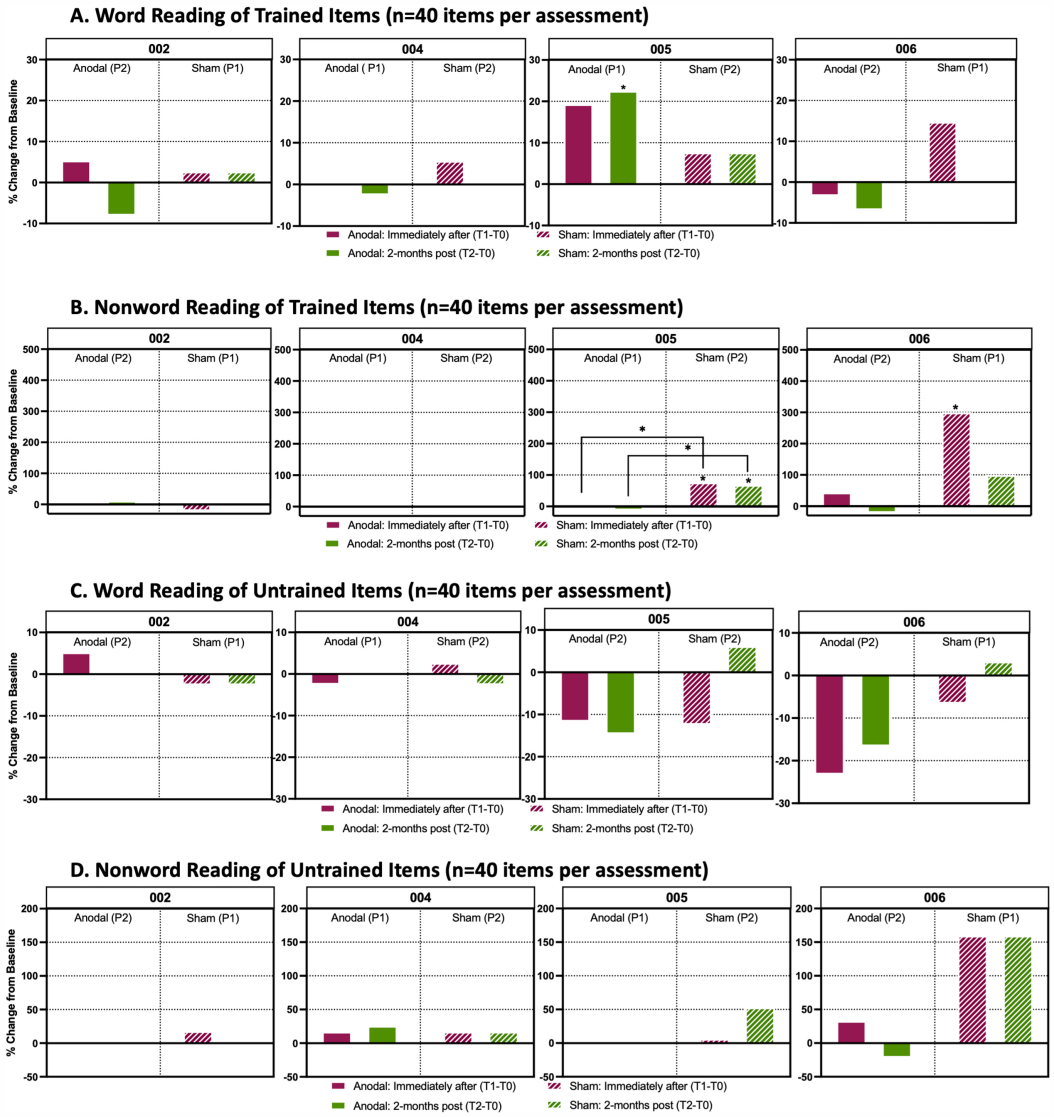
Analysis of word and non-word reading performance Percentage change from baseline in individual accuracy scores between timepoints in reading assessment. **(A)** Word reading from a list (*n* = 40 items per timepoint assessment) that participants were trained with during active anodal HD-tDCS (Anodal) or sham HD-tDCS (Sham) stimulation sessions. **(B)** Non-word reading task prepared from a list (n = 40 items per timepoint assessment) that participants were trained to during anodal treatment sessions. **(C)** Word reading assessment of items (*n* = 40 items per timepoint assessment) that participants were untrained to. **(D)** Non-word reading of items (*n* = 40 items per timepoint assessment) that participants were untrained to. Solid boxes indicate the change in performance from baseline when participants received anodal stimulation, whereas the stripped boxes indicate performance change from baseline when participants received sham. The maroon boxes identify the percent change from baseline immediately after the final stimulation session (T1-T0) and the green boxes show the percent change from baseline 2-months after the final stimulation session (T2-T0). P1 indicates that a participant received anodal or sham treatment during the first trial period; P2 indicates that a participant received the respective treatment during the second trial period after a 4-month washout following P1. An asterisk (*) above a box indicates that there was a significant change (*p* < 0.05) in performance between a participant’s baseline score and their assessments either immediately after treatment or 2-months post treatment. These assessments were analyzed using a Cochran *Q* and *post-hoc* McNemar test. An asterisk between two treatment periods indicates a significant change from baseline between anodal and sham treatment as determined by a Wilcoxon signed rank test.

No significant changes were observed on trained or untrained items for reading or repetition with sham or anodal treatment (Figures 5A,B, 6A–D).

#### Linguistic generalization and neuropsychological testing

For both sham (Period 1) and anodal (Period 2) treatments, P002 showed no significant change in scores on word rhyme matching, phonological short-term memory (Supplementary Figures S1A,C), letter or category fluency, picture naming, reading comprehension (Supplementary Figures S2A–D), or digit span (Supplementary Figure S3). Additionally, there were no marked differences in the participant’s performance on these tests between sham and anodal.

For the non-word rhyme matching assessment, P002 had a significant 25% decrease in % accuracy from baseline to T1 in Period 1 with sham; χ^2^(2) = 11.091, *p* = 0.004 and post-hoc McNemar (*p* = 0.008). When directly comparing stimulation conditions, there was a significantly improved performance from baseline to T1 with anodal compared to sham (Supplementary Figure S1B; *Z* = –2.568, *p* = 0.010; -25% sham vs. +14% anodal). Additionally, in treatment period 1, when P002 had received sham, there was an observable and significant decrease in performance on the Montreal Cognitive Assessment (MoCA) between baseline and T1, with the MoCA score falling from 20 to 12 (40% decrease); χ^2^(2) =8.167 *p* = 0.017 and post-hoc McNemar test *p* = 0.016 (Supplementary Figures S2E, S5E).

#### Discourse analysis

For both sham and anodal, P002 showed no significant change in scores from baseline in propositional idea density, words per minute, or syntactic complexity. Participant 002 showed a decrease in total words spoken over time (Supplementary Figure S4B), but the percent change between anodal and sham was not significantly different.

### Participant 004

#### Trained/untrained treatment items

P004’s non-word repetition showed little change throughout the study, with no significant changes in trained or untrained items for either sham (Period 2) or anodal (Period 1) conditions (Figures 5A,B). Word and non-word reading also showed no notable changes between sham versus anodal for either trained or untrained lists (Figure 6). Overall, P004 maintained a relatively high performance (60-80% accuracy) throughout the study.

#### Linguistic generalization and neuropsychological testing

For both sham and anodal stimulation conditions, P004 showed no significant change in scores from baseline in the performance of word and non-word rhyme matching, phonological short-term memory (Supplementary Figure S1), letter and category fluency, picture naming, reading comprehension, MoCA (Supplementary Figure S2), and digit span assessments (Supplementary Figure S3). Overall, P004’s performances showed only minor change between the two trial periods with no significant or notable changes across timepoints.

#### Discourse analysis

For both anodal and sham, P004 showed a decrease in idea density over time; however, the percent change from baseline to T2 in sham was significantly worse than the percent change in the anodal condition in idea density (Supplementary Figure S4A; *t* = –15.2, *p* = 0.04; – 15.3 sham vs. + 1.2% anodal). P004 showed an increase in total words spoken and words per minute overtime in both conditions (Supplementary Figures S4B,D), but the percent change between tDCS conditions was not significantly different. The participant showed a positive percent change in lexical diversity, being measured as the ratio of unique lexical items divided by the total number of words, in both conditions (Supplementary Figure S4C); the percent change was significantly greater for anodal compared to the sham condition (*t* = –31.3, *p* = 0.02; sham +4.4% vs. anodal +27%).

### Participant 005

#### Trained/untrained treatment items

P005 received anodal treatment in Period 1 and sham treatment in trial Period 2. Repetition of trained non-words after anodal stimulation significantly increased at T2 compared to T0 [χ^2^(2) = 8.778, *p* = 0.012 and *post-hoc* McNemars test *p* = 0.026; +21%]. There was also a trend toward increased performance at T1 (Figure 5A; *p* = 0.092). No changes in the untrained repetition of non-words were found between HD-tDCS conditions or across timepoints. Although both sham and anodal conditions produced >20% positive change from baseline at T1, these results were not significant after correcting for multiple pairwise comparisons; for anodal χ^2^(2) =6.333, *p* = 0.042 and post-hoc McNemar *p* = 0.114 (Figure 5B).

In reading performance, there was a significant increase in accuracy on trained words at T2 after anodal treatmentcompared to baseline (Period 1, Figure 6A; χ^2^(2) =9.556, McNemar test *p* = 0.03; +17%), and there was a trend toward increased accuracy at T1 (McNemar’s test *p* = 0.070; +14%). With sham HD-tDCS, performance for trained non-words was significantly improved at both T1 and T2 (Period 2; Figure 6B). There was a + 26% increase from baseline to T1: χ^2^(2) =11.375, McNemar’s test p = 0.012, and a + 23% increase from baseline to T2, McNemar’s test *p* = 0.022. These changes were significantly greater for sham when directly comparing T1-T0 and T2-T0 performances than anodal (T0-T1: Z = -2.353, *p* = 0.038, +26% sham vs-6% anodal; T0-T2: Z = -2.270, *p* = 0.023, +23% sham vs-8% anodal). For untrained items (words and non-words), reading performance was relatively stable, with no significant changes at T1 or T2 or between sham or anodal.

#### Linguistic generalization and neuropsychological testing

No changes were significant for word rhyme matching, phonological short-term memory, letter and category fluency, picture naming, reading comprehension, digit span, and MoCA tests. However, the performance on *non-word* rhyme matching did show a significant difference between HD-tDCS conditions from baseline toT1 with sham indicating greater improvement compared to anodal (*Z* = –2.558, *p* = 0.022, +17% sham vs-17% anodal) (Supplementary Figure S1B).

#### Discourse analysis

The recording of the speech sample from T2 after sham condition was lost due to technical difficulties, and so we do not have a complete data set. For anodal, no significant changes were found in any of the connected speech measures.

### Participant 006

#### Trained/untrained treatment items

Repetition of untrained non-words remained relatively stable at T1 and T2 for both sham (Period 1) and anodal (Period 2). Repetition of trained non-words improved with sham at both T1 and T2 timepoints (Figure 5A;. T1-T0: χ^2^2) = 15.739, McNemar’s test *p* = 0.019, +23%; T2-T0: McNemar’s Test *p* = 0.002, +31%). When directly comparing changes between sham and anodal, there was significantly larger increase from baseline to T2 (T2-T0) with sham than anodal HD-tDCS (Z = –3.024, *p* = 0.004, +31% sham vs. –5% anodal).

#### Linguistic generalization and neuropsychological testing

Reading of trained non-words was also significantly improved with sham from baseline to T1 (Figure 6B; χ^2^(2) = 10.500, McNemar’s test *p* = 0.008, +22%). Reading of trained or untrained words and untrained non-words did not change. There was trend toward improved reading of untrained non-words with sham [Figure 6D; χ^2^(2) = 7.11, McNemar’s test: p(T1-T0) =0.057, +20%; p(T2-T0) =0.079, +20%].

MoCA performance at baseline was significantly decreased at period 2 compared to period 1 (score of 3 from 9 respectively) as determined by Cochran Q test (Supplementary Figure S5E). No significant changes were noted in word or non-word rhyme matching, phonological short-term memory, letter or category fluency, picture naming, reading comprehension, or digit span with sham or anodal tDCS, or across timepoints.

#### Discourse analysis

For both sham and anodal, P006 showed general decreases over time in idea density, total words spoken, words per minute, and syntactic complexity (Supplementary Figures S4A,B,E). There was an increase in lexical diversity over time for both sham and anodal tDCS, likely due to the decrease in total words (Supplementary Figure S4C). For idea density, the percent increase with was significantly greater with sham than anodal tDCS (Supplementary Figure S4A; *t* = 267.1, *p* = 0.002; + 44.9% sham vs –10.5% anodal); no other comparisons for percent change in other discourse measures were significant.

### Group-level results

P001 initially left the study due to unrelated medical issues and later rejoined the study to completion as P006. P001 completed all neuropsychological and imaging procedures before withdrawing, and all were repeated upon re-enrollment almost a year later. P003 left the study after providing consent, but prior to the initiation of any stimulation procedures.

Study completion rate was 100%. This sample size of four is large enough to potentially exclude completion rates of 56% or lower in future trials based on a 90% exact binomial confidence interval (0.56–1).

Declines in MoCA averaged 4 points (range 1–6) in 3 of our participants between the baseline (T0) timepoints of the two treatment cycles, whereas one participant experienced a 3-point increase (P005). Declines in MoCA scores measured mid-way through the washout period (2-months post-intervention) averaged 2.5 points. For visualization of participant cognitive trajectories demonstrated through MoCA performance, see Supplementary Figure S5E.

Given the small sample size, the preliminary nature of our study, and the inability to perform a formal between-group statistical analysis, we set out to compare the total number of significant results in within each treatment condition (Anodal vs. Sham); a modest, but useful exploratory approach to help identify trends and patterns in the data (Tsapkini et al., 2014) (Supplementary Tables S2, S3). We found more significant improvements in the sham group (*n* = 9) compared to anodal HD-tDCS (*n* = 5). To compare the overall performance across both tDCS conditions, we counted and aggregated all statistically significant results. We found more significant results favoring sham (*n* = 9, +28% sham vs –5% anodal) compared to those favoring the anodal (*n* = 5, –1% sham vs. +26% anodal) condition (Supplementary Tables S2, S3). In an attempt to account for regression to the mean, we conducted a separate count excluding statistically significant outliers that could plausible be explained by a lower pre-treatment performance. In this case, the total number of significant results shifted in favor of anodal HD-tDCS (*n* = 2, +11% sham vs. +20% anodal) when compared to sham (*n* = 0) (Supplementary Figure S5B P005 and 006; Supplementary Figure S5C P006; Supplementary Figure S5D P005). Of note, the only two significant declines that were observed occurred under the sham condition (Supplementary Tables S3A,B). For connected speech, more significant improvements favored anodal (*n* = 2, + 14.1% anodal vs-5.45 sham) compared to sham (n = 1, + 44.9% sham vs-10.5% anodal) tDCS (Supplementary Figures S4A,C for P004).

Based on pre-treatment scores, participants were grouped as “high-performers” (002 and 004) and “low performers” (005 and 006). This grouping emerged from data inspection and averaging of baseline scores (Supplementary Figures S5A–D) High-performers average language task pre-treatment scores were 78.8 and 70.7% (74% total average). Low-performers average pre-treatment scores were 54.9 and 47.1% (51% total average). Of the post-treatment statistically significant changes that were observed, ‘low-performers’ (005 and 006) had only statistically significant score increases whereas P002, who was a ‘high-performer’, had two significant declines from pre-treatment performance in the non-word rhyming task and the MOCA. P004, also a ‘high-performer ‘, saw no statistically significant changes.

### fMRI results

The phonological connectome (Figure 2C) was calculated for each session (T0 and T1) and the percent change in connectivity was computed for both the anodal and sham conditions. No node was significantly different between anodal and sham treatments after correcting for multiple comparisons (pmin = 0.3). There was no apparent trend within participants. P005 had strong positive and negative changes in connectivity during the anodal condition. P006 had a modest increase in percent change in connectivity for left intra-hemisphere and left–right inter-hemisphere connections.

The percent change in connectivity between the TPJ and inferior parietal ROI (Figure 2E) were calculated for both the anodal and sham conditions. P006 had the greatest percent change in connectivity between anodal (3.9 %) and sham (0.89 %) for the left hemisphere. P005 had the greatest percent change in connectivity between anodal (1.3%) and sham (–0.59%) for the right hemisphere (Figure 2E). A group-level paired t-test was performed between anodal and sham percent change in connectivity between the inferior parietal and TPJ ROIs for both the left and right hemispheres. We found no significant difference in percent change in connectivity between anodal and sham for the left (*T*-value = 1.6, *p*-value = 0.33 corrected), and right (*T*-value = 1.9, *p*-value = 0.16 corrected) hemisphere.

### MEG results

#### Magnitudes of task-related evoked responses

All participants showed increased magnitudes of evoked responses at the source level within the left temporal–parietal region values in the post-anodal stimulation condition at T1 (Figure 3C). There was a decrease in source-level response magnitudes for three out of four participants for the post-sham condition. Comparison of post-anodal and post-sham conditions indicated increased response magnitudes across all participants. The largest increase was in P005, who had a 68.6% rise in response magnitudes. This was followed by P002 (37.6%), P006 (20.4%), and P004 (9.7%) as shown in Figure 3D. A summary of these findings and a comparison between the anodal and sham conditions are detailed in Supplementary Table S1.

#### LI analysis

For T0 vs. T1 comparisons, 3 out of 4 participants showed an increased relative LI for the anodal condition and the same 3 participants showed a decrease for the sham condition. Specifically, P002 (12.2%), 004 (13.5%), and 005 (10.6%) showed elevated LI values with anodal tDCS. P006 showed a 7% decrease in LI also with anodal tDCS. With sham tDCS, P002 (2.1%), P004 (–1.9%), and P005 (–3.2%) displayed minimal LI changes (< 5%), while P006 exhibited a decrease of-16.9% (Figure 4B).

Supporting evidence for the involvement of the left temporoparietal network during the word recognition task was provided by the source locations at the peak LI interval, observed in both the pre-and post-sham, and pre-and post-anodal conditions (Figure 4C). Notably, all participants showed more left-lateralized LI during the anodal compared to the sham tDCS (004: 15.49%, 005: 13.9%, 002: 10.0%, and 006: 9.8%), indicating the potential effectiveness of anodal tDCS in promoting phonological processing toward the left hemisphere (Figure 4D). A summary of the LI findings and a comparison between the anodal and sham tDCS are detailed in Supplementary Table S1.

## Discussion

In this case series study, we used a randomized, double-blind, sham-controlled, crossover design to examine the tolerability and feasibility of HD-tDCS to the posterior temporoparietal cortex, paired with phonological language training for patients with lvPPA. This case series is, to our knowledge, the first study to examine the feasibility of a 4-month washout period following HD-tDCS treatment cycle, in addition to the use of two different functional neuroimaging techniques (i.e., rsfMRI and MEG) to elucidate the neural underpinnings of tDCS. A secondary aim of this study was to gather preliminary efficacy data on changes in language, global cognition, brain resting-state functional connectivity, and task-related responses.

All four participants completed our study procedures in their entirety, including tDCS indicating that our approach is feasible. TDCS was well-tolerated by all 4 participants, with reports of only mild and self-resolving adverse events occurring without any lasting complications. Both participants and experimenters were successfully blinded, with accuracies below chance for participants in guessing the correct tDCS conditions. For experimenters, this accuracy was above chance but was not statistically significant. Finally, all participants completed the four rsfMRI and MEG scans that were required for this study.

Our findings related to the safety, feasibility, and blinding of HD-tDCS to the left temporoparietal cortex add to the growing literature, suggesting safe use of HD-tDCS in patients with lvPPA. Prior two studies used a frontotemporal montage different from the parietotemporal montage used in the current study: one participant in Unal et al. (2020), and eight participants in Nissim et al. (2022). This is an important distinction for feasibility and blinding assessment, as more posterior electrical stimulation montages have been linked to more intensive dizziness and pressure sensation, likely due to involvement of the vestibular nerve (Matsumoto and Ugawa, 2017). While these sensations were reported with the use of transcranial Alternating Current Stimulation (tACS), the perceived stimulation intensity and overall side-effects are reported to be higher with tDCS than tACS and so feasibility and blinding needed to be evaluated for posterior tDCS (Murray et al., 2023; Tavakoli and Yun, 2017). Additionally, we focused on a specific variant of PPA, the logopenic variant, as prior evidence suggests that the magnitude, duration, and generalization of outcomes with different stimulation targets differ when stratified for PPA variants (Coemans et al., 2021).

We successfully implemented a 4-month washout period, as no participants were lost to follow up and all completed the planned anodal and sham tDCS sessions, language assessments, MRI, and MEG assessments within the study timeline. The washout period in our study was 4 weeks longer than the next longest washout period used in prior PPA studies (Gervits et al., 2016; McConathey et al., 2017; Themistocleous et al., 2021). Our study is in support of the feasibility of longer washout periods, and we hypothesize that a longer washout period could help minimize carryover and ceiling effects related to delivering the next stimulation condition too soon. Notwithstanding this potential benefit, interval neurodegeneration and subsequent cognitive decline occurring within this same timeframe remain a major concern, as it would preclude participants serving as their own control. In a progressive disorder like lvPPA, worsening performance could mask stimulation benefits. This latter concern seems tangible when we look at the clinically significant changes in global cognition scores experienced by our participants (Krishnan et al., 2017; Wirth et al., 2022). For this reason and despite its feasibility, it might not be favorable to use a longer washout period. But even shorter washout periods are not ideal, given that a clinically meaningful cognitive decline was also seen at an earlier timepoint, mid-way through the washout period. While the use of crossover designs could still be favored to help with recruitment and to increase sample sizes, more comprehensive longitudinal assessments should be incorporated in future studies (Haslam and Prasad, 2018).

For this preliminary study in the ‘phonological’ variant of PPA, we focused on the targeting and engagement of the ‘phonological loop ‘by training non-word repetition which assesses the entire input–output phonology system and works as a type of constraint-induced therapy (Pillay et al., 2017).

We do recognize that moving forward the intervention should be modified to include a more established approach with more efficacy data. The training stimuli should be chosen to reflect individuals’ own personal and environmental factors in order to increase their life participation. Ideally, in a future clinical trial a certified Speech Language Pathologist should deliver the intervention, and home practice should be monitored for fidelity (Henry et al., 2013; Jokel et al., 2014; Rogalski and Khayum, 2018).

Multimodal imaging methods have shown additive effects supporting distinct brain-behavior associations and the combination of the high temporal resolution of MEG with the high spatial resolution of fMRI could prove complementary in this area of research (Grummich et al., 2006; Hall et al., 2014). MEG is a valuable tool to demonstrate neurophysiological signatures in each PPA syndrome (Ranasinghe et al., 2017) and the combination with fMRI could enhance its ability to predict the longitudinal course of the disease (Engemann et al., 2020).

With only four participants, our study is underpowered, limiting the reliability of behavioral and neural findings. There was inconsistent improvement across participants and tasks, with unclear predictors of response. Therefore, changes could represent individual variability rather than treatment effects and this could undermine conclusions regarding HD-tDCS efficacy. Despite the exploratory nature of the study, we ventured to identify trends that can help inform the design of future larger trials.

We made a preliminary and exploratory attempt to understand if cognitive status could influence outcomes. ‘Low’ pre-treatment performers appeared to experience more behavioral gains compared to ‘High’ performers, who saw no gains or loses. This suggests an inverse association between pre-treatment scores and the direction of changes after tDCS, potentially due to regression toward the mean, a well-known confound in behavioral research. Another possibility is that tDCS resulted in more gains in more cognitively impaired participants with poorer pre-treatment scores. This observation is consistent with several prior tDCS studies in PPA (Gervits et al., 2016; Hung et al., 2017; McConathey et al., 2017; Tsapkini et al., 2014). Additionally, greater brain atrophy, more loss of function and poorer baseline language scores have all been linked to greater potential for functional improvement (Coemans et al., 2021; de Aguiar et al., 2020; McConathey et al., 2017; Wang et al., 2013). As previously reported by McConathey et al. (2017), HD-tDCS may benefit patients more at later stages in the disease process. Future studies should seek to minimize regression toward the mean by averaging multiple baseline observations, or alternatively, by tailoring the task difficulty to patients’ impairment levels using adaptive procedures (Barnett, 2004).

Factors affecting stimulation delivery could also influence the variability in response across participants. For example, greater cortical atrophy could mean increased CSF of the target region, which may result in a more diffuse current. For this reason, cortical dosage can vary widely across individuals, and 2 mA at the scalp may yield significantly different effective doses at the stimulation target. Not using electrical field modeling to determine if the current dose was administered as intended was a limitation of our study and should be addressed by future trials.

Although contrary to the prevailing evidence, a true inferiority of anodal tDCS over sham cannot be completely ruled out. Although not demonstrated in clinical trials or practice, it is essential to entertain the possibility of non-invasive brain stimulation techniques, such as HD-tDCS, promoting the induction of maladaptive plasticity (Crosson et al., 2019). Of note, no instances of generalization to untrained items were observed in this study. This study, albeit quite small, focuses on a single PPA variant and stimulation of a focal and atrophic brain area. In prior studies, more subtle and idiosyncratic effects might have remained hidden due to the inability to stratify by PPA variant due to small sample sizes. In previous studies of PPA, MCI, and healthy aging populations, the behavioral gains from anodal tDCS have been associated with decreases in functional connectivity (Ficek et al., 2019; Meinzer et al., 2013, 2015). This seems to contradict a facilitatory role associated with anodal tDCS, and as a result, an alternative mechanism has been proposed that suggests that anodal tDCS is associated with introduction of noise and subsequent network ‘decoupling’ in PPA (Ficek et al., 2019). In our case, we cannot rule out a more typical excitatory effect of anodal HD-tDCS that perpetuated or enhanced already existing patterns of aberrant functional hyperconnectivity.

Our preliminary interpretation of the MEG and rsfMRI data appear to be incongruent with the previous findings indicating decline in functional connectivity (Ficek et al., 2019; Meinzer et al., 2013; Meinzer et al., 2015). Instead, our findings suggest more excitatory effects of anodal tDCS compared to sham. Specifically, all participants had increased left hemispheric MEG evoked response amplitudes and consequently higher laterality indices (LI) after anodal compared to sham conditions, indicating greater responsivity of the left temporoparietal network (Figure 4D). P004, the participant with no statistically significant behavioral changes, showed the highest LI and the lowest response amplitude change with anodal tDCS, possibly due to a ceiling effect. Whereas P005, the participant with the highest number of significant improvements, showed the greatest response amplitude change, the strongest anodal > sham increases in functional connectivity in the phonologic connectome, and the strongest inferior parietal-TPJ increases in functional connectivity within the right hemisphere. These findings provide initial support for smaller LI change, larger temporoparietal activation, and increased connectivity within the phonologic network as potential neural correlates of a clinical response to tDCS. While neural activation changes were observed, their relevance to behavioral outcomes is tenuous and this is a clear limitation of our study. Ultimately, whether these neural correlates represent a deleterious versus a beneficial compensatory adaptation should be the subject of further investigation.

With the advent of tDCS as a promising therapeutic and rehabilitation tool in both post-stroke and neurodegenerative aphasias, it seems likely that these conditions will differ in their optimal neuromodulation approach as a direct result of their distinct underlying pathophysiologic mechanisms: stimulation of spared perilesional and contralateral areas in the former and disease-specific and growingly inefficient brain regions in the latter. This premise is strongly supported by existing literature, with stroke-related studies showing major neuroanatomical reorganization after the initial injury (Abel et al., 2015; Dancause et al., 2005) and PPA studies revealing a relatively rapid neurodegenerative process that leaves no space for major reorganizational changes of brain functional networks (Rogalski et al., 2011; Sonty et al., 2007). Sonty et al. (2003) is a good illustration of this point. In this study fMRI was used to compare signal changes during phonological and semantic language tasks. While PPA participants showed longer reaction times and reduced accuracy on language tasks, they continued to show a relatively unchanged pattern of activation within the classical language regions, with only minor differences compared to healthy controls. A later study by the same group (Sonty et al., 2007) reproduced similar findings using a semantic task, with PPA participants showing a similar left-lateralized pattern of activation when compared to controls in the inferior frontal gyrus (IFG), temporoparietal cortex (TPC), anterior cingulate, and supplementary motor area.

We believe this small study contributes modestly to our understanding of tDCS delivered to atrophic and peri-atrophic areas in PPA and complements existing body of literature to guide future larger-scale clinical trials. Improvements in written-naming abilities in patients with PPA have been demonstrated after stimulation to the left IFG in an oral/written-naming task (Tsapkini et al., 2018). Participants with nfvPPA, whose main site of atrophy is the stimulated left IFG, seemed to benefit most from that treatment (Coemans et al., 2021). Cotelli et al. (2016) focused on patients with nfvPPA and targeted the DLPFC with stimulation. The IFG was most likely affected by stimulation, given the use of a conventional tDCS montage (Coemans et al., 2021). Alternatively, functional targeting approaches (using task-based fMRI activation maps to identify stimulation targets) can increase efficacy of tDCS, particularly for more focal HD-tDCS applications. These approaches circumvent regional inefficiencies related to functional and anatomical decay in atrophic and periatrophic regions (Coemans et al., 2021). Recent studies show that patients with PPA undergo some level of functional reorganization during disease progression, with generation of new hubs and the loss of old ones (Mandelli et al., 2018; Tao et al., 2020). Mandelli et al. (2018) reported that many of these new critical nodes were found in the right hemisphere. Consistent with these findings, P005 who was a ‘responder’ with most improvements also experienced the greatest increase in functional connectivity within the right phonologic network. This suggests that stimulation of the right hemisphere could play an important therapeutic role given our phonological treatment in patients with lvPPA. Understanding these functional and neurophysiological changes will prove essential in the identification of variant-specific as well as personalized stimulation targets for improving tDCS efficacy.

## Conclusion

Our study findings support the feasibility and tolerability of daily applications of HD-tDCS to the left temporoparietal cortex, coupled with a phonological language treatment protocol in patients with lvPPA. A long washout period of 4 months is feasible in crossover studies, but not ideal given the concerns about progressive cognitive decline in PPA. A multimodal approach to functional neuroimaging using rsfMRI and MEG is possible in PPA and holds promise in helping us better understand the neural underpinnings of tDCS.

No firm conclusions can be drawn regarding the efficacy of HD-tDCS because of the large variability in response and the small sample size. But adding to the existing literature, our findings suggest that pre-treatment language performance and disease severity can be potential predictors of tDCS response. Evoked response amplitudes from the MEG data were the strongest and consistent across all four participants, indicating greater activation of the temporoparietal network targeted by anodal tDCS. Increases in resting-state functional connectivity with anodal tDCS appeared stronger in ‘responders’ than non-responders. The behavioral implications of the use of HD-tDCS in lvPPA should be the subject of future larger trials.

## Data Availability

The raw data supporting the conclusions of this article will be made available by the authors, without undue reservation.
