# Supplementary material for: Behavioral and neural effects of temporoparietal high-definition transcranial direct current stimulation in logopenic variant primary progressive aphasia: a preliminary study
Source: Front Psychol. 2025 Feb 25;16:1492447. doi: 10.3389/fpsyg.2025.1492447 (PMC11893574; doi:10.3389/fpsyg.2025.1492447)
Supplement: Supplementary file 1 [file Data_Sheet_1.docx]

**C. Repetition of Trained Nonwords (n=48 items per assessment)**

**B. Reading of Trained Nonwords (n=40 items per assessment)**

**A. Reading of Trained Words (n=40 items per assessment)**

**D. Nonword Rhyming Task (n=36 items per assessment)**

**Supplementary Figure 5: Cognitive assessments in which at least one statistically significant finding occurred.** Change in individual participant language task performance at three assessment timepoints (T0=baseline; T1=Immediately after stimulation session 10; T2=2-months post stimulation) and between the treatment periods in which participants received either anodal HD-tDCS (Anodal) or sham treatment (Sham). (**A**) **Reading of trained words** (n=40 items per assessment); (**B) Reading of trained nonwords** (n=40 items per assessment); (**C**) **Repetition of trained nonwords** (n=48 items per assessment); (**D) Rhyming of nonwords** (n=36 items per assessment; Pillay et al. 2017). **(E) Montreal Cognitive Assessment** ( n = 30 items per assessment). Solid lines between timepoints indicate participant performance when anodal stimulation was given, whereas interrupted lines indicate when sham was used. **P1** indicates the first trial treatment period; **P2** indicates the second treatment period after a 4-month washout period following P1. A black asterisk (*****) between two timepoints indicates a significant change from baseline within a treatment period that was assessed using a Cochran Q test and post-hoc McNemar test. A red asterisk (*) between two timepoints indicates a significant change between P1 and P2 baseline performances. A red hash (**#**) is used to indicate a significant change in performance between anodal and sham treatments as determined by a Wilcoxon signed rank test. Grayed graphs show significant score changes that cannot be explained by lower baseline performance scores (both under anodal HD-tDCS).

**E. Montreal Cognitive Assessment (n=30 items per assessment)**
