# Supplementary material for: Behavioral and neural effects of temporoparietal high-definition transcranial direct current stimulation in logopenic variant primary progressive aphasia: a preliminary study
Source: Front Psychol. 2025 Feb 25;16:1492447. doi: 10.3389/fpsyg.2025.1492447 (PMC11893574; doi:10.3389/fpsyg.2025.1492447)
Supplement: Supplementary file 2 [file Data_Sheet_2.docx]

| **A.** |  |  |  |
| --- | --- | --- | --- |
| **Participant** | **Anodal** | **Sham** | **Anodal > Sham** |
| 002 | 30.3 | -7.3 | 37.6 |
| 004 | 28.8 | 19.1 | 9.7 |
| 005 | 46.1 | -22.5 | 68.6 |
| 006 | 21.0 | 0.6 | 20.4 |
| **B.** | | | |
| **Participant** | **Anodal** | **Sham** | **Anodal > Sham** |
| 002 | 12.2 | 2.1 | 10.1 |
| 004 | 13.5 | -1.9 | 15.4 |
| 005 | 10.6 | -3.2 | 13.8 |
| 006 | -7.0 | -16.9 | 9.9 |

**Supplementary Table 1: MEG Task Source Magnitudes and Laterality Indices following anodal and sham brain stimulation**. **A**) Changes in MEG task magnitudes near the stimulation site, **B**) Laterality values in the temporoparietal network. These findings align with the results depicted in **Figure 3**.

| **Participant**  **(A) Nonword Repetition**  **Supplementary Table 2: Trained and Untrained Task Performance** | **Treatment Order** | **Immediately After (T1-T0)** | | | | **2-months Post (T2-T0)** | | | | | |
| --- | --- | --- | --- | --- | --- | --- | --- | --- | --- | --- | --- |
|  |  | **Trained** | | **Untrained** | | **Trained** | | | | **Untrained** | |
|  |  | **HD-tDCS** | **Sham** | **HD-tDCS** | **Sham** | **HD-tDCS** | | **Sham** | | **HD-tDCS** | **Sham** |
| **002** | Sham; Anodal | **n.s.** | **n.s.** | **n.s.** | **n.s.** | **n.s.** | | **n.s.** | | **n.s.** | **n.s.** |
| **004** | Anodal; Sham | **n.s.** | **n.s.** | **n.s.** | **n.s.** | **n.s.** | | **n.s.** | | **n.s.** | **n.s.** |
| **005** | Anodal; Sham | **n.s.** | **n.s.** | **n.s.** | **n.s.** | **↑**  **(p=0.026)** | | **n.s.** | | **n.s.** | **n.s.** |
| **006** | Sham; Anodal | **n.s.** | **↑**  **p=0.019** | **n.s.** | **n.s.** | **n.s.** | **anodal<sham**  **p=0.004** | | **↑**  **p=0.002** | **n.s.** | **n.s.** |

**(C) Nonword Reading**

| **Participant**  **(B) Word Reading** | **Treatment Order** | **Immediately After (T1-T0)** | | | | **2-months Post (T2-T0)** | | | |
| --- | --- | --- | --- | --- | --- | --- | --- | --- | --- |
|  |  | **Trained** | | **Untrained** | | **Trained** | | **Untrained** | |
|  |  | **HD-tDCS** | **Sham** | **HD-tDCS** | **Sham** | **HD-tDCS** | **Sham** | **HD-tDCS** | **Sham** |
| **002** | Sham; Anodal | **n.s.** | **n.s.** | **n.s.** | **n.s.** | **n.s.** | **n.s.** | **n.s.** | **n.s.** |
| **004** | Anodal; Sham | **n.s.** | **n.s.** | **n.s.** | **n.s.** | **n.s.** | **n.s.** | **n.s.** | **n.s.** |
| **005** | Anodal; Sham | **n.s.** | **n.s.** | **n.s.** | **n.s.** | **↑**  **p=0.032** | **n.s.** | **n.s.** | **n.s.** |
| **006** | Sham; Anodal | **n.s.** | **n.s.** | **n.s.** | **n.s.** | **n.s.** | **n.s.** | **n.s.** | **n.s.** |

| **Participant** | **Treatment Order** | **Immediately After (T1-T0)** | | | | | | **2-months Post (T2-T0)** | | | | | |
| --- | --- | --- | --- | --- | --- | --- | --- | --- | --- | --- | --- | --- | --- |
|  |  | **Trained** | | | | **Untrained** | | **Trained** | | | | **Untrained** | |
|  |  | **HD-tDCS** | | **Sham** | | **HD-tDCS** | **Sham** | **HD-tDCS** | | **Sham** | | **HD-tDCS** | **Sham** |
| **002** | Sham; Anodal | **n.s.** | | **n.s.** | | **n.s.** | **n.s.** | **n.s.** | | **n.s.** | | **n.s.** | **n.s.** |
| **004** | Anodal; Sham | **n.s.** | | **n.s.** | | **n.s.** | **n.s.** | **n.s.** | | **n.s.** | | **n.s.** | **n.s.** |
| **005** | Anodal; Sham | **n.s.** | **anodal<sham**  **p=0.038** | | **↑**  **p=0.012** | **n.s.** | **n.s.** | **n.s.** | **anodal<sham**  **p=0.023** | | **↑**  **p=0.022** | **n.s.** | **n.s.** |
| **006** | Sham; Anodal | **n.s.** | | **↑**  **p=0.008** | | **n.s.** | **n.s.** | **n.s.** | | **n.s.** | | **n.s.** | **n.s.** |

McNemar’s test, two- tailed, results for individual participant change in performance between treatment timepoints (T1-T0=between baseline and immediately after treatment session 10; T2-T0=between baseline and 2-months post stimulation). A green upward arrow (**↑**) indicates that a participant’s score significantly (p<0.05) increased between baseline and either T1 or T2 following treatment with either HD-tDCS or sham. Wilcoxon signed-rank, two-tailed, test compares participants score change from baseline between HD-tDCS and sham. Significant result cells were highlighted with the condition having a greater increase indicated with a > or <. **A.** results for the nonword repetition language task; **B.** results for the word reading task; **C.** results for the nonword reading task.

| **Participant** | **Treatment Order**  **(A) Nonword rhyming**  **Supplementary Table 3: Other Language and Cognitive Task Performance** | **Immediately After (T1-T0)** | | | | | | **2-months Post (T2-T0)** | |
| --- | --- | --- | --- | --- | --- | --- | --- | --- | --- |
|  |  | **HD-tDCS** | | | **Sham** | | | **HD-tDCS** | **Sham** |
| **002** | Sham; Anodal | **n.s.** | **anodal>sham**  **p=0.020** | | | | **↓**  **p=0.008** | **n.s.** | **n.s.** |
| **004** | Anodal; Sham | **n.s.** | | | **n.s.** | | | **n.s.** | **n.s.** |
| **005** | Anodal; Sham | **n.s.** | | **anodal<sham**  **p=0.022** | | **n.s.** | | **n.s.** | **n.s.** |
| **006** | Sham; Anodal | **n.s.** | | | **n.s.** | | | **n.s.** | **n.s.** |

**(B) Montreal Cognitive Assessment**

| **Participant** | **Treatment Order** | **Immediately After (T1-T0)** | | **2-months Post (T2-T0)** | |
| --- | --- | --- | --- | --- | --- |
|  |  | **HD-tDCS** | **Sham** | **HD-tDCS** | **Sham** |
| **002** | Sham; Anodal | **n.s.** | **↓**  **p=0.008** | **n.s.** | **n.s.** |
| **004** | Anodal; Sham | **n.s.** | **n.s.** | **n.s.** | **n.s.** |
| **005** | Anodal; Sham | **n.s.** | **n.s.** | **n.s.** | **n.s.** |
| **006** | Sham; Anodal | **n.s.** | **n.s.** | **n.s.** | **n.s.** |

| **Participant** | **Treatment Order** | **Immediately After (T0-T1)**  **(C) Word Rhyming; Category Fluency; Letter Fluency; Phonological Short-Term Memory; Picture naming; Reading Comprehension; Digit Span: Forward, Backward, Sequence** | | **2-months Post (T0-T2)** | |
| --- | --- | --- | --- | --- | --- |
|  |  | **HD-tDCS** | **Sham** | **HD-tDCS** | **Sham** |
| **002** | Sham; Anodal | **n.s.** | **n.s.** | **n.s.** | **n.s.** |
| **004** | Anodal; Sham | **n.s.** | **n.s.** | **n.s.** | **n.s.** |
| **005** | Anodal; Sham | **n.s.** | **n.s.** | **n.s.** | **n.s.** |
| **006** | Sham; Anodal | **n.s.** | **n.s.** | **n.s.** | **n.s.** |

McNemar’s test, two- tailed, results for individual participant change in performance between treatment timepoints (T1-T0=between baseline and immediately after treatment session 10; T2-T0=between baseline and 2-months post stimulation). A red downward arrow (**↓**) indicates that a participant’s score significantly (p<0.05) declined between baseline and either T1 or T2 following treatment with HD-tDCS or sham. Wilcoxon signed-rank, two-tailed, test compares participants score change from baseline between HD-tDCS and sham. Significant results were highlighted, with the condition having a better task performance indicated with a > or <. **A.** nonword rhyming task, which had participants with significant changes either from baseline or between treatment conditions; **B.** Montreal Cognitive Assessment, which had a participant with significant change from baseline performance; **C.** word rhyming, category fluency, letter fluency, phonological short-term memory, picture naming, reading comprehension, digit span forward, digit span backward, digit span sequence, and Montreal cognitive assessment (MoCA), all of which had non-significant task performance changes throughout the study.
