# Supplementary figures and images for: Behavioral and neural effects of temporoparietal high-definition transcranial direct current stimulation in logopenic variant primary progressive aphasia: a preliminary study
Source: Front Psychol. 2025 Feb 25;16:1492447. doi: 10.3389/fpsyg.2025.1492447 (PMC11893574; doi:10.3389/fpsyg.2025.1492447)

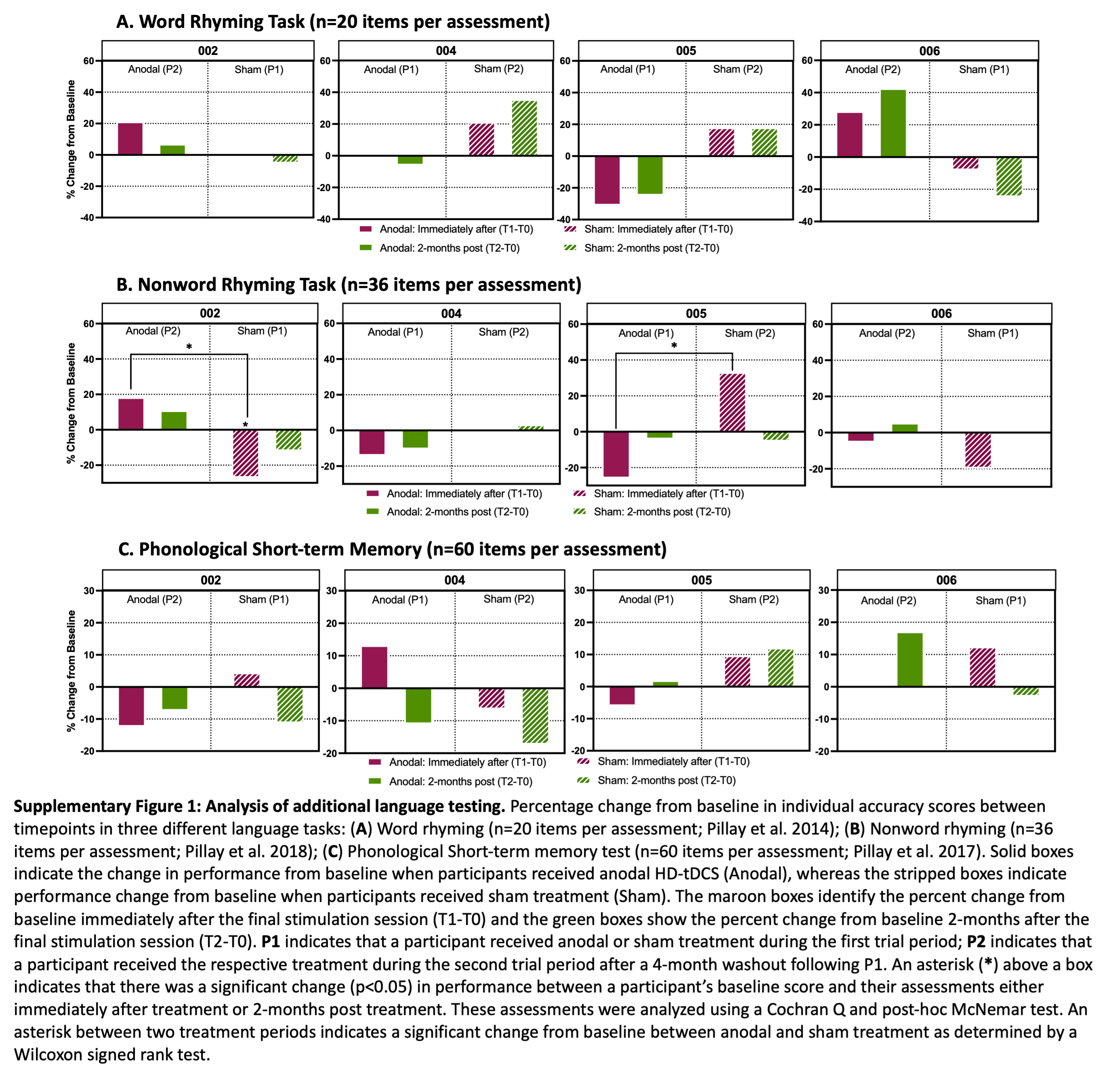

Supplement: Supplementary file 3 [file Image_1.jpeg]

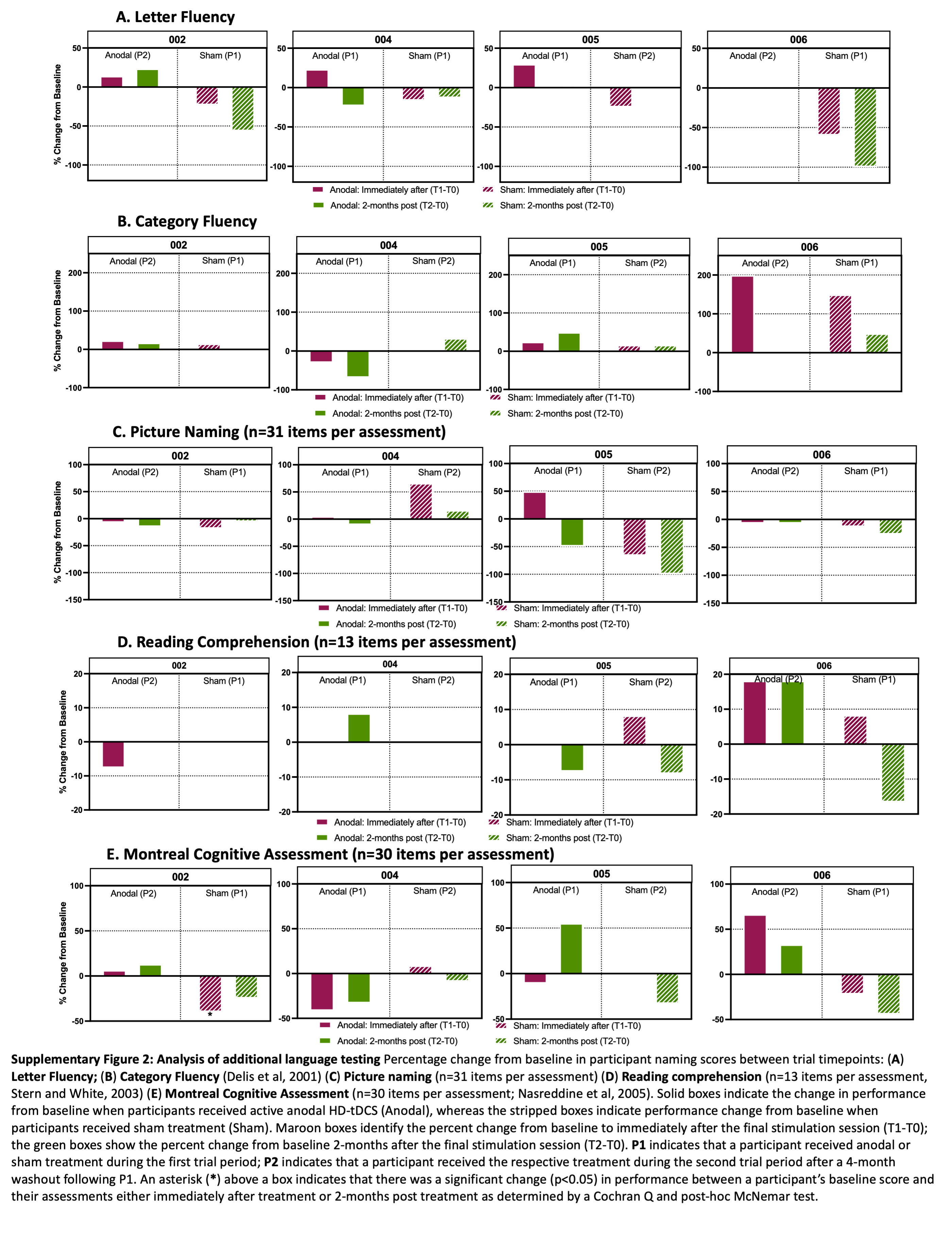

Supplement: Supplementary file 4 [file Image_2.jpeg]

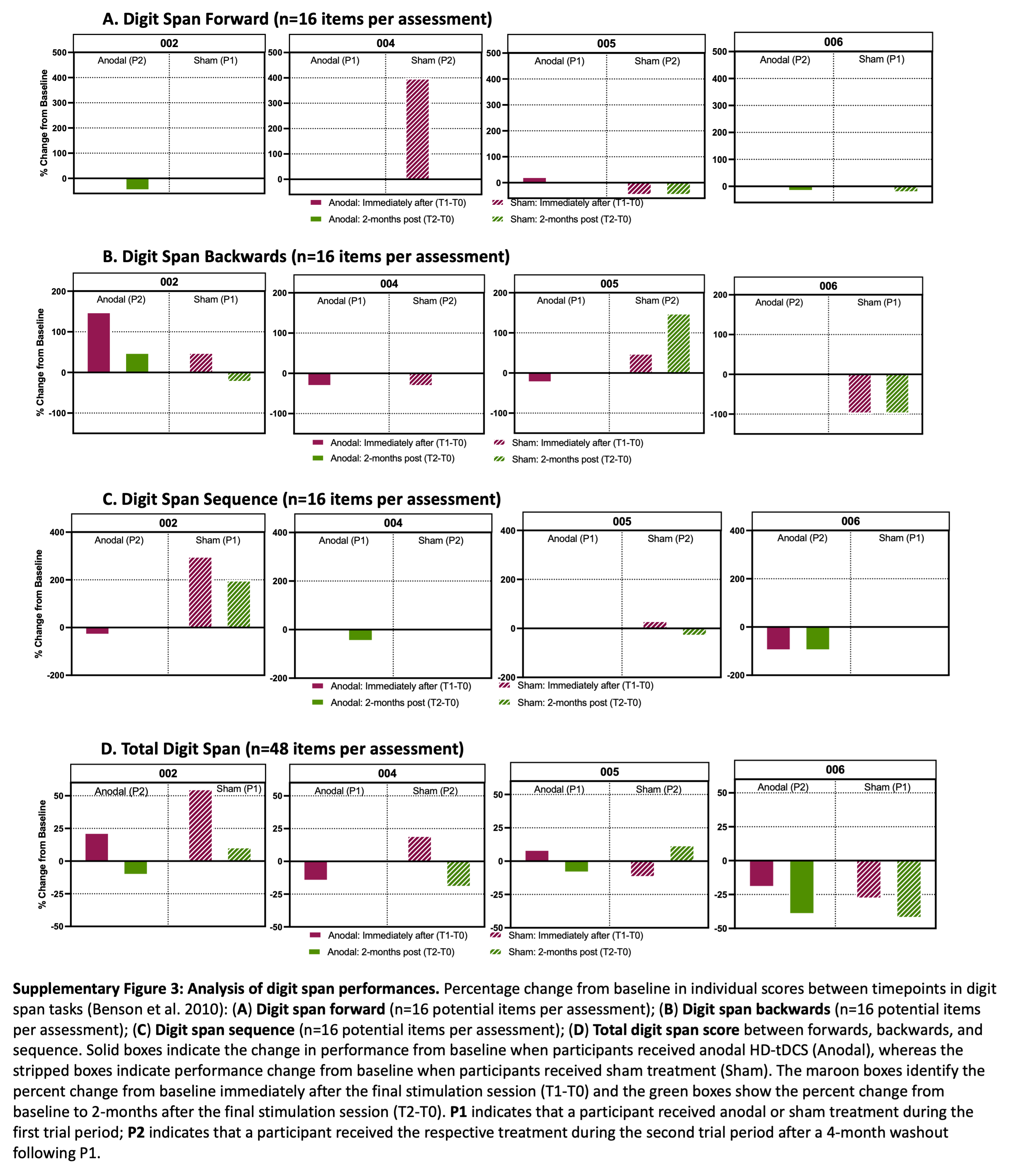

Supplement: Supplementary file 5 [file Image_3.jpeg]

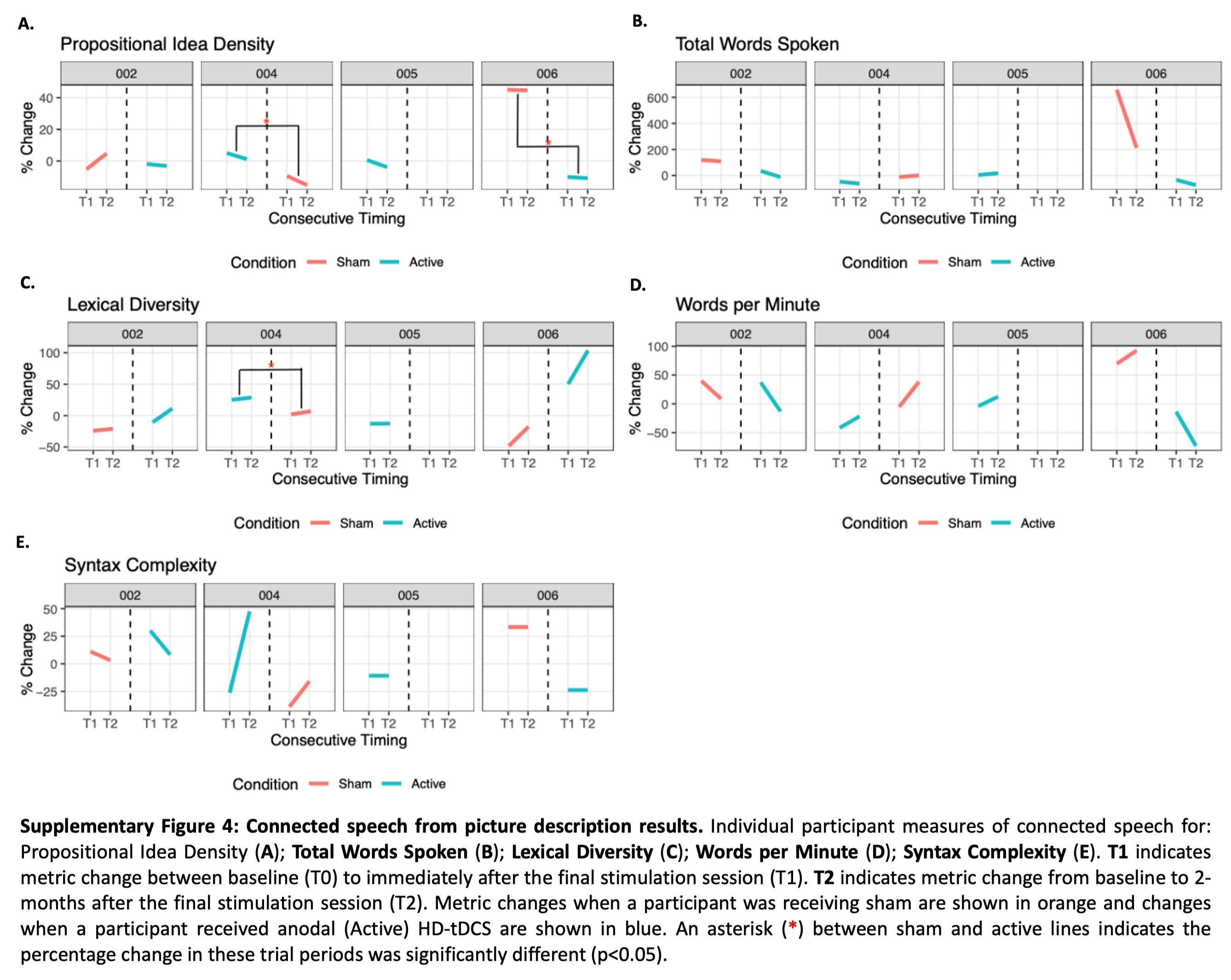

Supplement: Supplementary file 6 [file Image_4.jpeg]
